# Tannic acid-loaded hydrogel coating endues polypropylene mesh with hemostatic and anti-inflammatory capacity for facilitating pelvic floor repair

**DOI:** 10.1093/rb/rbac074

**Published:** 2022-09-26

**Authors:** Chenghao Wu, Zixuan Zhou, Xi You, Yi Guo, Ping Chen, Huaifang Li, Xiaowen Tong

**Affiliations:** Department of Obstetrics and Gynecology, Tongji Hospital, Tongji University School of Medicine, Shanghai 200065, People’s Republic of China; Department of Burn Surgery, The First Affiliated Hospital of Naval Medical University, Burn Institute of PLA, Shanghai 200433, People’s Republic of China; Department of Obstetrics and Gynecology, Tongji Hospital, Tongji University School of Medicine, Shanghai 200065, People’s Republic of China; Department of Obstetrics and Gynecology, Tongji Hospital, Tongji University School of Medicine, Shanghai 200065, People’s Republic of China; School of Life Sciences and Technology, Tongji University, Shanghai 200092, People’s Republic of China; Department of Obstetrics and Gynecology, Tongji Hospital, Tongji University School of Medicine, Shanghai 200065, People’s Republic of China; Department of Obstetrics and Gynecology, Tongji Hospital, Tongji University School of Medicine, Shanghai 200065, People’s Republic of China

**Keywords:** tannic acid, hydrogel, polypropylene mesh, pelvic floor repair

## Abstract

The application of polypropylene mesh (PPM) in pelvic organ prolapse (POP) treatment was severely limited by the complications associated with PPM, such as mesh exposure, chronic inflammatory reactions and postoperative hematoma. This study applied a method of fabricating a hydrogel-mesh complex (PPM + TA@GelMA) to cross-link tannic acid (TA) directly with Methacrylate Gelatin (GelMA) hydrogel and thus to form a coating for PPM. This one-step coating modification improved the hydrophilicity and cyto-compatibility of PPM. The hemostatic effect of PPM+TA@GelMA was confirmed through tail amputation test. Through the defect tissue repair experiments *in vivo*, it was proved that PPM+TA@GelMA had effects of anti-inflammation and promoting tissue repair and regulated the M2 subtype macrophages polarization for tissue repair. The TA-loaded hydrogel coating endued PPM with multiple functions. It is believed that the novel hydrogel-mesh complex and its fabrication method will have great significance in basic research and clinical application.

## Graphical Abstract

**Figure:**
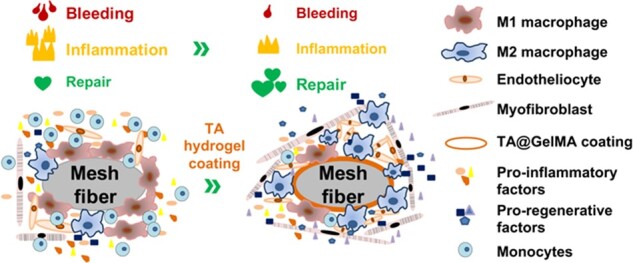

## Introduction

Polypropylene mesh (PPM) is made of polypropylene (PP) monofilaments, which has high chemical stability and relatively good mechanical property as the standard material. PPM is used to treat pelvic organ prolapse (POP) clinically, which significantly improves anatomic correction in prolapsed patients [1, 2]. However, the occurrence of complications due to mesh exposure seriously affects the life quality of patients [3, 4]. Pathological evidences suggest that the vast majority of complications are related to acute or chronic inflammation induced by heterologous mesh materials [4, 5]. The main influencing factor of exposure is the poor histocompatibility of the mesh causing the inflammatory response around the mesh [1, 2, 6]. A risk of postoperative hematoma has been reported recently [7]. Large-sample studies show hematoma or bleeding has a high occurrence perioperatively [8] and is one of the major complications within 2 months after surgery [9].

Inhibition of postoperative inflammatory response and extracellular matrix (ECM) degradation is the key to reducing mesh exposure and corrosion [1, 10]. Various cells and cytokines are involved in various acute and chronic inflammatory processes after mesh implantation [1, 11]. Macrophages can be divided into two main subtypes, M1 and M2. M2 macrophages are anti-inflammatory and promote tissue repair [12].

While large amounts of biomaterials were recently investigated for replacing PPM in surgical treatment of POP [13–15], the therapeutic effect of most biomaterials has not been verified by clinical trials. A few of clinical trials have not even shown good long-term anatomic outcomes in clinical studies [16, 17] and parts of them are just in their infancy, lacking further rigorous and high-quality clinical evidences [18].

Thus, the coating modification of PPM is an option for the secondary development of the clinically applied PPM. Hachim *et al*. [19] fabricated a PPM mesh coating for sustained release of IL-4, which could effectively promote the M2 polarization of macrophages at the repair site in animals, and inhibit the occurrence of inflammation. Silver nanoparticles coated PPM mesh [20], platelet-rich plasma (PRP) gel coating [21], ECM gel coating [22] and collagen coatings [11, 23] have been investigated on the antibacterial properties, inflammation inhibition and biocompatibility modification of meshes respectively. However, few studies have improved PPM with multiple properties through simple and effective methods.

Biomaterials with various components and nanostructures such as hydrogels have been applied for studies of bone tissues [24–26], nerve [27, 28], cartilage [29, 30] and soft tissues [15, 31] regeneration. Methacrylate Gelatin (GelMA) is a commonly applied hydrogel derived from gelatin and methacrylic anhydride (MA) with a variety of biomedical applications such as tissue repair and drug delivery [32]. It needs to be cross-linked under photosensitive conditions, and the initiator of cross-linking is a synthesized chemical reagent, which may be cytotoxic. In the fields of tissue repair and regeneration, an increasing amount of Chinese herbal medicines, including Lawsone [33] and Puerarin [15, 34], were loaded into bioactive materials for introducing more properties. Likewise, TA is a kind of polyphenolic compound extracted from tea leaves [35]. TA can be cross-linked with a variety of polymer hydrogels through hydrogen bonding interactions to form hydrogels, enhance the mechanical strength of hydrogels [36] and endue hydrogels with intrinsic anti-inflammatory, antioxidant, and antibacterial properties [37–39]. TA can also be employed to crosslink the chitosan nanofibers for the wound healing applications as a simple and effective technique [40].

The purpose of this research is to form an anti-inflammatory and hemostatic coating for PPM with TA-loaded GelMA hydrogel, which will promote pelvic floor tissue repair and regeneration.

## Materials and methods

### Materials and reagents

TA, gelatin, MA, lithium phenyl-2,4,6-trimethylbenzoylphosphinate (LAP) and trypsin were from Sigma-Aldrich (St Louis, US). PPMs were self-woven according to the previous study [41]. BARD meshes were purchased from BARD Corporation (USA). Live/Dead Viability/Cytotoxicity Kit and DAPI solution were from Invitrogen (USA). BCA Protein Assay Kit, Cell Counting Kit-8, DAB Immunohistochemistry Color Development Kit, ECL luminescence reagent, Hematoxylin–eosin (HE) staining kit, protease inhibitor and RIPA Lysis Buffer were from Sangon Biotech (China). Masson Staining Kit, Sirus Staining Kit and immunohistochemical (IHC) staining kits, RNA Extraction Kit were from Solarbio Life Science (China). PrimeScript RT reagent kit was from TaKaRa Bio Inc (Japan). Matrigel was from BD Biosciences (USA).

Anti-Human antibodies included anti-Collagen I (ab260043), anti-Collagen IIIα1 (ab184993), anti-MMP2 (ab92536), anti-MMP3 (ab52915), anti-MMP9 (ab76003), anti-TIMP2 (ab180630), anti-TIMP3 (ab39184), anti-GAPDH (ab8245) from Abcam (UK). Anti-Rat antibodies included anti-Collagen I (ab260043), anti-Collagen III (ab7778), anti-MMP2 (ab92536), anti-MMP3 (ab52915), anti-MMP9 (ab76003), anti-TIMP2 (ab180630), anti-α-SMA (ab7817), anti-CD86 (ab238468), anti-CD206 (ab64693), anti-TGF-β1 (ab215715) and anti-GAPDH (ab8245) were from Abcam (UK) and anti-TIMP3 (5673S) was from CST (USA). Anti-mouse antibody was APC/Cyanine7 anti-CD11b (101226) and FITC anti-CD206 (C068C2) from Militenyi Biotech (Germany). Second antibodies were Goat anti-Mouse IgG (H + L) Secondary Antibody, HRP (31430) and Goat anti-Mouse IgG (H + L) Secondary Antibody, HRP (32460) from ThermoFisher (USA). All primers (Supplementary Table S1) were synthesized by Sangon Biotech (China). Other materials or reagents without specific description were obtained from Beyotime Biotech (China).

### Cell culture

Human fibroblasts (HFBs) were a gift from Prof. Shichu Xiao [42]. L929 and RAW 264.7 macrophage cell lines were from Meilian Biotech Company (China) and National Collection of Authenticated Cell Cultures (China), respectively. Human umbilical vein endothelial cells (HUVECs) were from the China Center for Type Culture Collection (China). All cell lines were cultured with high glucose DMEM medium supplemented with 10% FBS (Gibco, USA) and 100 units/ml Penicillin/Streptomycin Solution (Sangon Biotech, China) under 5% CO_2_ at 37°C in a humidified incubator (ThermoFisher, USA).

### Fabrication of TA@GelMA hydrogel coating

#### Preparation of GelMA hydrogel

Ten grams of gelatin were added to 100 ml of PBS, swollen at room temperature (RT) for 1 h, and stirred at 60°C until completely dissolved. MA was added to the gelatin solution at a rate of 1 ml/min dropwise. After reacting at 50°C for 3 h, the reaction solution was added with 400 ml of PBS at 50°C, then kept into a dialysis bag of 8–14 kDa, and dialyzed in PBS for 1 week. The dialysate was centrifuged at 3000 rpm for 10 min, and the supernatant was placed at −80°C overnight and vacuum freeze-dried to obtain gelatin methacrylamide (GelMA). GelMA materials were dissolved in PBS and filtered through a 0.22 μm filter to prepare GelMA hydrogel.

#### Formation of TA@GelMA hydrogel coating

The 100 μl GelMA solution was pippeting on PPM, and hydrogel coating was formed. Through introducing sterile TA solution, without ultraviolet (UV) and LAP (crosslink initiator), in <1 min, GelMA hydrogel (total volume: 100 μl) crosslinked and formed TA@GelMA hydrogel coating of PPM. PPM with GelMA hydrogel coating and PPM with TA@GelMA hydrogel were named as PPM + GelMA and PPM + TA@GelMA, respectively. Freeze-dried PPM + GelMA and PPM + TA@GelMA were applied for *in vivo* investigations and wet ones for cellular experiments.

### Characterization of hydrogel-mesh complexes

#### Scanning electron microscope

PPM, PPM + GelMA and PPM + TA@GelMA were lyophilized for 48 h after crosslinking. After gold spray, the surface morphology of the hydrogel-mesh complexes was observed under scanning electron microscope (SEM). Three random areas were selected for capturing pictures.

#### Water contact angle

All samples were prepared with strips and placed on glass slides. A droplet of 4 μl ddH_2_O was dropped on the surface of the sample. Contact angles between droplets and surface of samples were recorded. All samples were measured by three times and the mean was acquired.

#### Water retention ratio

The water retention ratios (WRRs) of the hydrogel coatings were evaluated by exposing the mesh-hydrogel complexes (size: 1.5 cm × 1.5 cm, thickness = 0.60 mm) to the environment with 30% relative humidity at RT (about 25°C). And their weight (W_t_; t represents recording hours) was recorded using an electronic scale (XPR2U/AC, Mettler Toledo, USA) at regular intervals over 96 h (every 2 h in the first 24 h, every 4 h in the second 24 h and every 8 h in the remaining 48 h). The calculation equation: WRR (%) = (*W*_0_ − *W*_t_)/*W*_0_ × 100%. *W*_0_ represents the initial weight of complexes (*t* = 0 h).

Finally, the mesh-hydrogel complexes were lyophilized for SEM to observe the coating morphology after the 96-h water loss.

#### Swelling ratio assay

The swelling property of the hydrogel coatings was analyzed by incubating the mesh-hydrogel complexes (size: 1.5 cm × 1.5 cm, thickness = 0.60 mm) to PBS at 37°C. At regular intervals, the weight of the swelling complexes (*W*_t_) was measured, and the swelling ratio (SR) was calculated by: SR (%) = (*W*_t_ − *W*_0_)/*W*_0_ × 100%. *W*_0_ represents the initial weight of complexes (*t* = 0 h).

Since the wet hydrogel coatings might slightly absorb a small volume of water, changes in the total weight of the wet mesh-hydrogel complexes could not be obviously observed. Both freeze-dried and wet mesh-hydrogel complexes were evaluated for their swelling behavior.

#### *In vitro* degradation assay

Due to the non-negligible hydro-scopicity of wet hydrogel coatings, the weight changes of wet mesh-hydrogel complexes did not reflect the authentic degradation property. The degradation property of hydrogel coatings was investigated by measuring the initial freeze-dried weight (W_i_) of complexes and the freeze-dried weight after incubating in 0.25% trypsin in PBS solution at each time points (*W_t_*). The degradation ratio (DR) was calculated by: DR (%) = (*W_i_* – *W_t_*)/*W_i_* × 100%.

#### Mechanical properties

##### Tensile strength test [43]

All samples (∼0.270 mm thickness) were cut into dumbbell-shaped splines (with 15 mm length and 10 mm width). Uniaxial tensile tests were conducted with a force test instrument (Hengyi Corporation, China) at the tensile speed of 10 mm/min at RT. The stress–strain curves were obtained and Young’ s modulus was calculated after three random tests.

##### Ball burst strength test [43]

The prolapse mechanism of POP was that pelvic organs were pushed to outlet of vagina by giant abdominal and pelvic stress due to severe cough and constipation. Ball burst strength test was used to detect the essential force to burst meshes. A 25 mm stainless-steel ball was positioned over the mesh (6 × 6 cm) and forced to rupture mesh at a constant rate of 100 mm/min. The ultimate burst strength was reported and recorded by a ball burst strength tester (SANSCM, China).

### *In vitro* cyto-compatibility assay

PPM, PPM with GelMA hydrogel or TA@GelMA hydrogel were placed on a 12-well plate and 20 000 HFBs were cultured on the surface. The cyto-compatibility of meshes or hydrogel-meshes was detected at 3 and 7 days by LIVE/DEAD Viability/Cytotoxicity Kit. Green and red fluorescence were observed under a fluorescence microscope (Leica, Germany).

### *In vitro* cell proliferation assay

Meshes or hydrogel-meshes were soaked in high glucose DMEM medium for 24 h and supernatants were collected and filtrated to culture HFBs. Cells were rinsed carefully three times by PBS. Then CCK8 solution was added to each well and incubated at 37°C for 3 h. Finally, the absorbance was measured at 450 nm wavelength by a microplate reader (SpectraMax M5, Molecular Devices, USA).

### POP animal model and surgical operation

As Supplementary Fig. S1a–d shows that the rat abdominal wall muscle defect model was established to mimic POP as reported [22]. Female Sprague-Dawley (SD) rats (weight range 180–250 g) were chosen and all procedures were strictly performed according to protocols approved by the Ethical Committee of Tongji Hospital of Tongji University. Anesthesia was induced and maintained with about 5% and 2–3% isoflurane respectively. Preoperative preparation included removing fur and sterilizing skin. After bilateral skin incision and dissection of all subcutaneous loose tissues, a 2 cm × 2 cm PPM or freeze-dried PPM + TA@GelMA overlaid a 1 cm × 1.5 cm (width × length, L × W) partial muscular defect with *transversalis fascia* intact randomly, which was sutured with 3-0 PROLENE^TM^ sutures interruptedly. Abdominal walls were closed, sterilized and bandaged finally. At each time point (different weeks: 1, 2, 3 and 8 weeks), five rats were sacrificed to explant mesh-tissue complexes for further histological analysis.

### Gross evaluation of tissue-mesh complexes

Macroscopic images were taken to evaluate the gross histological outcomes of implanted meshes (Supplementary Fig. S1e). Complications including exposition, abscess, infection and fold were observed.

### Hemostatic capability test

The hemostatic capability of mesh and hydrogel-meshes were evaluated by rail tail amputation model. Female SD rats (weight range 180–250 g) in each group for this assay were anesthetized with isoflurane and were cut ∼50% length of tail by surgical lancets. Then, different treatment groups including control (no treatment), gauze, PP mesh, freeze-dried PP mesh+GelMA and freeze-dried PP mesh+TA@GelMA were applied. In the meantime, both blood loss weight and hemostatic time were recorded.

### Histological and IHC staining

Mesh–tissue complexes were washed with PBS, fixed in 4% paraformaldehyde (PFA) overnight and embedded in paraffin after dehydration. Four micrometers thickness slices were sectioned and dewaxed for further histological staining.

#### H&E and Masson’s trichrome staining

H&E and Masson staining were in accordance with standard protocols of kits and samples were followed by dehydration of alcohol gradient. After air drying, the resin gel sealed slices. Histological and collagen deposition evaluation were conducted.

#### Sirius staining

Slices were stained by Sirius dye solution for 8 min, dehydrated by absolute ethanol three times, and subjected to xylene until transparent for 5 min. The resin gel sealed slices. Under a polarized light microscope, specific collagen types and distributions were identified. The thicker fibers were red in polarized light color, and the thinner ones were green.

#### Immunohistochemistry staining

IHC staining was conducted to evaluate the regeneration of muscle/fiber tissues and blood vessels and to distinguish macrophages phenotype. After deparaffinization, slides were stained according to protocols of IHC kits. The primary antibodies used were mouse anti-rat α-SMA, mouse anti-rat CD68 and rabbit anti-rat CD206 antibodies all at a dilution of 1:50. The secondary antibodies used were at a dilution of 1:100. The positive reactions were defined as those showing brown signals, observed at random and photographed.

### Biochemical experiments

#### Western blotting

Cellular samples were processed as we previously published. Tissue samples were cut into tiny pieces, homogenized until no visible solids and centrifuged to get supernatants. After determination of protein concentration by BCA method, 35 μg of protein samples were separated by SDS-PAGE. The primary antibodies including anti-Collagen I, Collagen III, MMP2, MMP3, MMP9, TIMP2, TIMP3 and GAPDH were all at a dilution of 1:1000. The secondary antibodies used were at a dilution of 1:5000.

#### Real-time quantitative polymerase chain reaction

Tissue samples were fully grinded in liquid nitrogen. The total RNA of cells or tissue samples was extracted with an RNA Extraction Kit and reverse transcribed into cDNA with a PrimeScript RT reagent kit. Then cDNA was performed real-time quantitative polymerase chain reaction (RT-qPCR) on an ABI Step One System for detecting target gene. GAPDH was used as the reference gene.

### Immunofluorescence cell staining, flow cytometry, transwell migration assay and tube formation assay

Supernatants from meshes or hydrogel-meshes soaking solution mentioned before were used for inducing RAW 264.7 macrophages polarization for 48 h.

For immunofluorescence cell staining, RAW 264.7 macrophages were cultured and induced in glass bottom dishes for confocal microscopy. After being fixed with 4% PFA, cells were permeabilized with 0.1% Triton X-100, blocked in blocking buffer and incubated with APC/Cyanine7 anti-CD11b and DAPI. Fluorescence images were taken under OLYMUPS confocal microscope (Japan).

For flow cytometry, a total of 10^6^ RAW 264.7 cells per group was suspended in PBS and incubated with FITC anti-mouse CD206. Fluorescence of cells was detected under BD FACSCanto flow cytometer (USA).

For transwell migration assay, a total of 10^5^ L929 cells were seeded without serum in the upper chamber, and supernatants were in the lower chamber. After 24 h of incubation, the upper chamber was fixed in 4% PFA and stained with 0.1% crystal violet. Migrated cells were counted for analysis.

For tube formation assay, 60 μl precooled Matrigel per well was coated on a 96-well plate and solidified at 37°C for 30 min. Then, HUVECs (5× 10^4^ cells/well) were cultured and incubated with the abovementioned supernatants. After incubating at 37°C for 5 h, tube-like structures were observed under an inverted optical microscope (Leica, Germany) and the total loop numbers were counted.

### Statistical analysis

All data were processed by GraphPad Prism 8.3.0, presented as mean ± standard deviation (SD) and analyzed by Student’s *t*-test or one-way ANOVA. *P <* *0.05, **P <* *0.01, ***P <* *0.001 and ****P <* *0.0001 represented different levels of statistical significance.

## Results and discussion

### Fabrication of TA@GelMA coating on PPM

**Figure 1.**
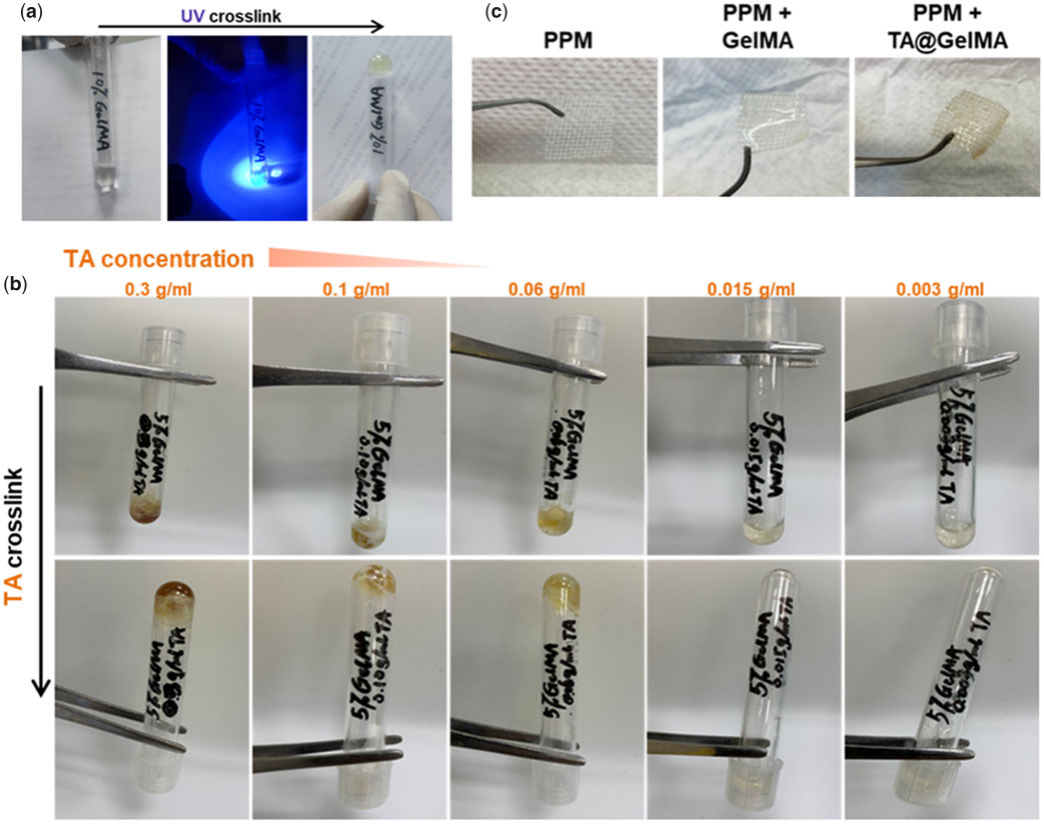
Fabrication of TA@GelMA + PPM. (**a**) Crosslinking GelMA hydrogel with UV; (**b**) crosslinking GelMA hydrogel with tannic acid (TA) to form TA@GelMA hydrogel with changing the concentration of TA; (**c**) representative images of polypropylene mesh (PPM), PPM with GelMA hydrogel coating and PPM with TA@GelMA hydrogel coating.

GelMA hydrogel was crosslinked through UV initiating in LAP solution (Fig. 1a) under darkness. Without UV and initiator, herein, TA extracted from tea was utilized for directly crosslinking with 5% (w/v) GelMA in PBS solution. It has been reported that TA could crosslink with hydrogels through hydrogen binding effects reported [35, 36] in different fields including wound repair [37, 38], clinical sutures [44] and refs [35, 45]. As presented in Fig. 1b, hydrogel was well formed when TA concentration is 0.06 g/ml; however, in higher concentration (0.3 g/ml, 0.1 g/ml) of TA, the GelMA protein was excessively coagulated, and well-distributed and translucent hydrogel was not formed. Obviously, hydrogel was not crosslinked in lower concentration. The PPM was woven with polypropylene monofilaments according to our previous studies [41]. GelMA and TA@GelMA hydrogels were successfully crosslinked on the PPM and fabricated coatings (Fig. 1c). PPM+TA@GelMA appeared tan due to the presence of TA.

The thickness of bare PPM was about 0.35 mm; the thickness of wet hydrogel-mesh complexes (size: 1.5 cm × 1.5 cm) was about 0.60 mm; the thickness of freeze-dried ones was reduced to about 0.50 mm.

### Characterization of meshes

**Figure 2.**
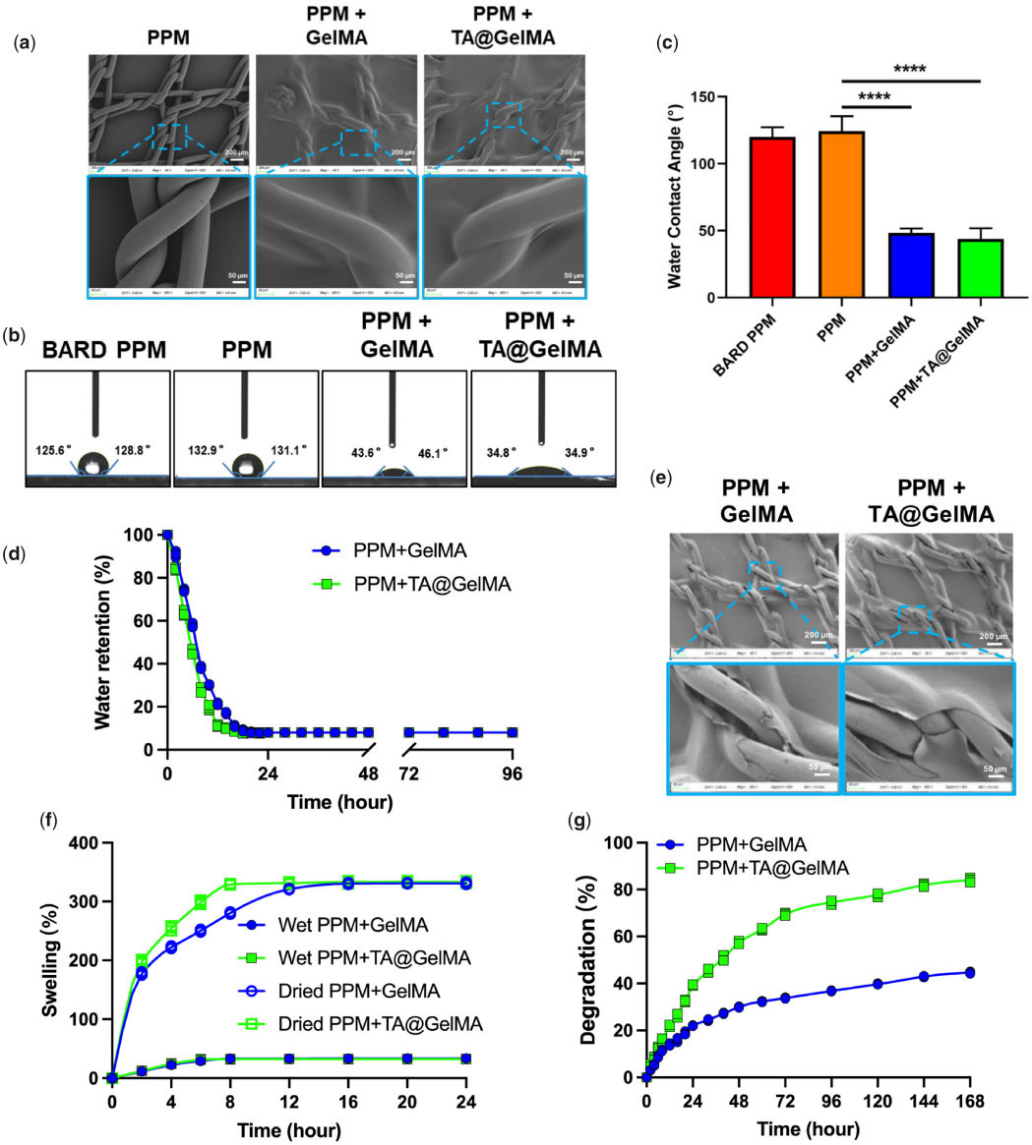
Characteristics of mesh or hydrogel-meshes. (**a**) Micro-morphological images of PPM, PPM + GelMA and PPM + TA@GelMA under SEM; (**b**) WCAs of mesh and hydrogel-mesh complexes; (**c**) calculated results of WCAs; (**d**) WRR of the hydrogel-meshes over time; (**e**) micro-morphological images of the hydrogel-meshes after water loss; (**f**) swelling test of the freeze-dried and wet hydrogel-meshes; (**g**) degradation test of the hydrogel-meshes. ****P <* *0.0001.

The micro-morphology of the surfaces of PPM or hydrogel-mesh complexes was observed through SEM. Compared with PPM group, there were hydrogel coatings wrapping the monofilaments, knots and pores in PPM+GelMA and PPM+TA@GelMA groups (Fig. 2a). The PPM is inert, hydrophobic and allows no attachment of cells [46], which hinders the integration of mesh with tissues and might be one of the reasons for the complications of mesh exposure. Biocompatibility could be improved by increasing the surface hydrophilicity of biomaterials [47, 48]. Through the detection of the water contact angle (WCA) between different groups, the results showed that the surface hydrophilicity of the PPM + GelMA and PPM + TA@GelMA groups was improved (Fig. 2b). BARD mesh, one of the most used meshes in the 2010s, was as a negative control. The WCAs of BARD PPM, PPM, PPM + GelMA and PPM + TA@GelMA groups were 120.2° ± 7.0°, 124.3° ± 11.24°, 48.28° ± 3.4°, 43.9° ± 7.9°, respectively. There was no significant statistical difference between PPM and BARD PPM. In contrast, both PPM + GelMA and PPM + TA@GelMA groups showed significant statistical differences from the PPM group in the hydrophilic angle (Fig. 2c). A hydrophilic hydrogel coating formed on the surface of the mesh achieved the effect of material dressing modification, suggesting that hydrogel-meshes could provide a good wettable environment for cell colonization and growth and tissue remodeling.

The water retention, swelling and degradation properties of hydrogel-modified coatings could be characterized by the following several important assays. The water retention assay was performed to evaluate the water holding capacity of wet hydrogel coatings. As shown in Fig. 2d, the WRRs of both PPM + GelMA and PPM + TA@GelMA groups decreased rapidly in the first 24 h and maintained a steady status in the remaining hours. During the first 8 h, water molecules in the form of free water rapidly evaporated in both groups. Compared to GelMA hydrogel coating, TA@GelMA hydrogel coating showed a higher rate of water loss mainly due to the low cross-linking density. After the duration of rapid water loss, TA@GelMA hydrogel coating had a significantly lower reduction rate before reaching the steady status. In the end, no statistical differences were shown in the total water loss between two groups. The micro-morphology of dehydrated hydrogel-mesh complexes was evaluated under SEM (Fig. 2e). Overall, the hydrogel coatings became thinner in both groups after dehydration. Although parts of the coating in both groups began to crack at the knots, it could be clearly seen that the majority of monofilaments were still tightly wrapped by the hydrogel coating.

In this study, the swelling assays of wet and dried hydrogel-mesh complexes were performed in PBS at 37°C. Results in Fig. 2f showed that a higher rate of water uptake in the dried TA@GelMA hydrogel coating compared to the dried GelMA hydrogel and no significant differences were existed in the SRs between two wet hydrogel coating groups. Evidences [49, 50] demonstrated that the faster swelling capacity of hydrogels coating endowed the complexes to concentrate coagulation factors in shorter time, which was more conducive to hemostasis. The freeze-dried hydrogel-mesh complexes, which are more suitable for storage in vacuum packages compared to wet complexes, can absorb water molecules in a very short time and convert them into wet complexes better for cell attachment and proliferation.

*In vitro* degradation assays could mimic *in vivo* enzymatic biodegradation processes of collagen-derived hydrogel scaffolds [51]. In our study, the degradation percentage of TA@GelMA hydrogel coating at 1 week (168 h) was 84.00 ± 1.00%, which was significantly higher than that of GelMA hydrogel coating (44.63±0.40%, *P *<* *0.0001; Fig. 2g). During the early stage of hydrogel-complexes implantation, the degradation of TA@GelMA hydrogel coating would be gradually synchronous with remodeling and regeneration with new tissue, and TA was released for reducing and regulating the inflammatory immune cell infiltrated microenvironment induced by PPM implantation [1]. Conversely, slower biodegradation rate of GelMA hydrogel coating could lead to chronic inflammation and hinder the integration of tissues and PPM.

Additionally, the ideal pelvic floor reconstruction meshes [52] required excellent mechanical properties to maintain the integrity of the pelvic floor anatomy without organ prolapse under enormous pelvic and abdominal pressures. Therefore, a tensile test was performed to determine the tensile properties of the material and a burst test was performed to determine the maximum burst strength that would destroy the integrity of the mesh. Supplementary Figure S2a shows the stress–strain curves of different groups under uniaxial tension. The ultimate tensile strengths of the PPM, PPM + GelMA and PPM + TA@GelMA groups were 18.38 ± 0.16, 18.56 ± 0.60 and 18.01 ± 0.62 MPa, respectively. The Young’s modulus of the stress–strain curve was presented in Supplementary Fig. S2b. There was no significant statistical difference in the Young’ s modulus among the three groups. These results indicated that the hydrogel coating did not impair the tensile mechanical properties of meshes and hydrogel-meshes. POP is a disease in which pelvic organs descends due to the weakening or degeneration of the supporting tissues of the pelvic floor. Bulging of the pelvic organs resembled the onsets of abdominal wall hernias. Therefore, through the ball burst strength test (as shown in Supplementary Fig. S2c), the occurrence of POP could be simulated, and the ultimate load force required for bursting could be obtained. The burst strengths were 550.0 ± 10.00, 540.7 ± 6.03 and 548.0 ± 14.42 N with no significant difference (Supplementary Fig. S2d), indicating that the hydrogel coating did not alter the burst strength properties of meshes and hydrogel-meshes. Although studies were reported that TA could strengthen the mechanical properties of hydrogels [36], which could be neglected due to the strong mechanical properties of PPM, hydrogel did not affect the strength of the mesh itself. It was worth noting that some studies [15, 53, 54] had reported new materials for pelvic floor repair, such as hydrogels and electro-spun patches, etc. Although these biomaterials could degrade and promote tissue repair, the maintenance of their mechanical strength *in vivo* was still worth considering. Since PPM had strong mechanical properties, which were not altered by hydrogel coating **(**Supplementary Fig. S2), herein hydrogel coating was aimed to improve the cyto- and histo-compatibility of PPM, regulate immune response, and promote tissue remodeling and repair.

### *In vitro* bio-compatibility of meshes

**Figure 3.**
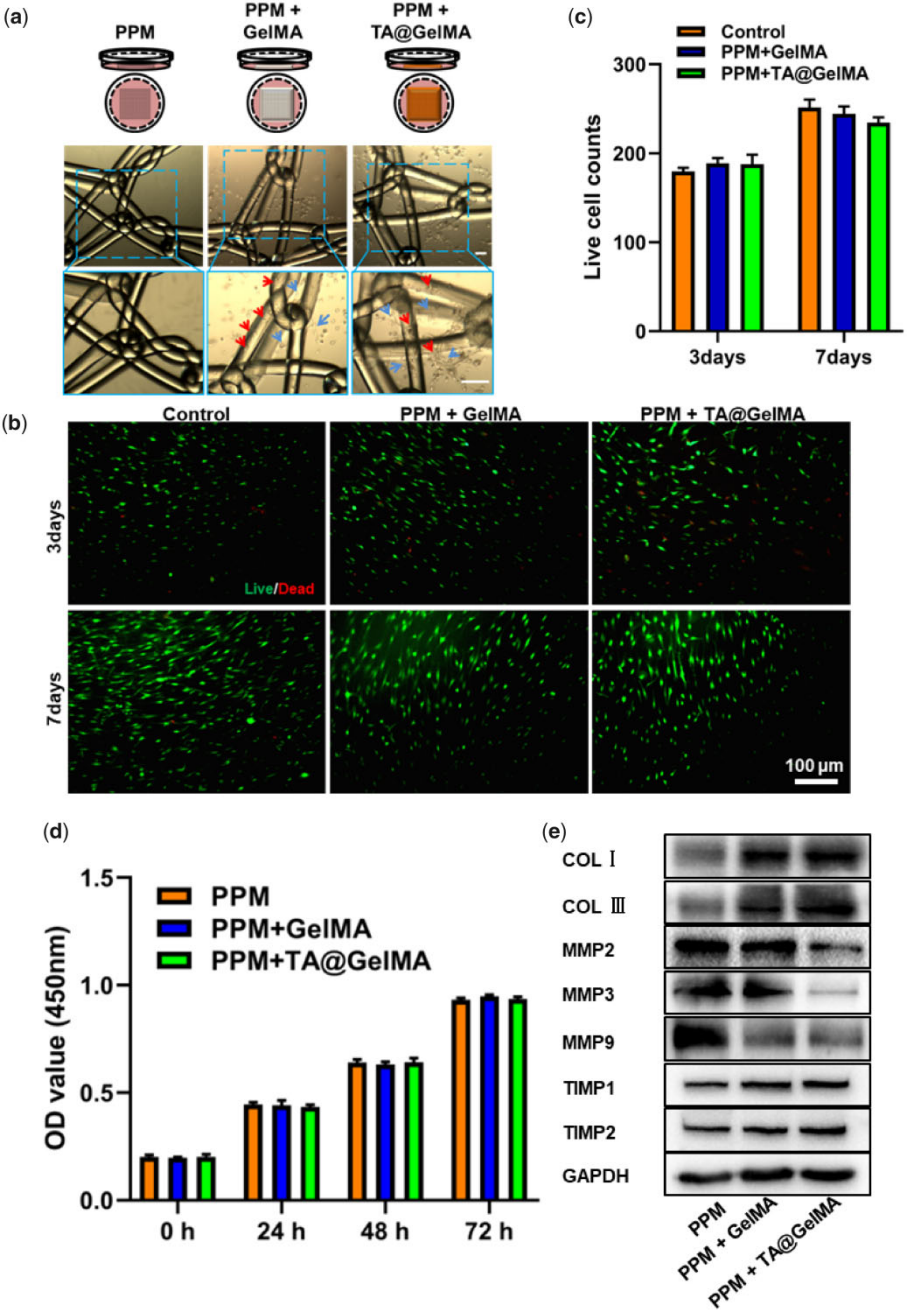
*In vitro* biocompatibility evaluation. (**a**) HFBs cultured with hydrogel-mesh composites. No cells attached on PPM fibers without hydrogel coating; (**b**) live/dead staining. Green fluorescent dots are alive cells and red fluorescent dots are dead cells; (**c**) live cell counts; (**d**) CCK-8 assay for proliferation of HFBs; (**e**) Western blotting results of Collagen I (COL I), Collagen IIIα1 (COL IIIα1), MMPs (MMP2, MMP3, MMP9), tissue inhibitor of metalloproteinase (TIMP2, TIMP2) and glyceraldehyde-3-phosphate dehydrogenase (GAPDH). scale bar, 100 μm.

Five percent of (w/v) GelMA solution was used to resuspend the HFBs cell pellet, and the resuspended solution was crosslinked on the PPM. After the hydrogel-mesh complexes were cultured in a complete medium for 48 h, the complexes were taken out and observed under an optical microscope. As shown in Fig. 3a, in the groups of hydrogel-mesh complexes, HFBs could be attached to the mesh monofilament fibers or distributed in the mesh pores. Figure 3b shows the live/dead staining results of HFBs cultured on the surface of cell-free hydrogel-mesh complexes for 3 and 7 days. And Control group represented HFBs cultured on the cell-culture dishes. At 3 days, several dead cells were visible in each group. At 7 days, fibroblasts in each group massively proliferated, and no obvious dead cells or few dead cells were observed. There was no statistically significant difference in the number of viable cells (the counts of green fluorescent cells) in each group at 3 and 7 days (Fig. 3c). Due to the uneven surface of the hydrogel-mesh complexes, the fluorescent cells could not be all observed at the same layer. For proliferation assay, HFBs were cultured with supernatants from different groups, and after 1, 3, 5 and 7 days, the cell proliferation in each group was assessed using the Cell Counting Kit-8 (CCK-8). As shown in Fig. 3d, the optical density (OD) value at 450 nm of each group increased with the number of days; there was no statistical difference in OD value among different groups at the same time point. The above results suggested that TA did not adversely affect the viability and proliferation of cells, which was consistent with the reported results [37]. The hydrogel coating could improve the hydrophobicity and poor biocompatibility of PPM, and promote the mesh-cell interaction as a bridge. Through Western blotting experiments, the protein expression related to ECM remodeling in fibroblasts was explored. Western blotting results showed that the expression of collagen related proteins (COL I, COL III) was up-regulated, and ECM degradation related proteins, such as matrix metalloenzymes (MMPs) (MMP2, MMP3, MMP9), were down-regulated, while matrix metalloproteinase inhibitors (TIMP2, TIMP3) up-regulated (Fig. 3e). Fibroblasts in PPM + GelMA and PPM + TA@GelMA groups expressed more repair-related proteins and fewer MMPs playing roles in the ECM degradation pathway. The above results suggested that PPM + TA@GelMA had good cyto-compatibility and promoted fibroblasts to participate in tissue repair and remodeling *in vitro*.

### Hemostatic assay

In order to place PPMs in the correct anatomical position, both transvaginal and abdominal pelvic reconstruction surgeries require surgeons to dissect diffusely and sometimes dissociate tissues blindly, which may cause blood vessel perforation and micro-vessel damage [8]. Perioperative bleeding and postoperative hematoma will definitely increase the occurrence of transfusion and adverse events in PPM-based surgeries [9]. Besides mesh exposures, excessive intraoperative bleeding could increase the risk of postoperative pain [55]. For some adverse events, some patients could get relief with proper management, but some of whom would need mesh removal or re-operation, indicating meshes are not integrated with tissues and clinical outcomes are opposite to the purpose of using mesh for reconstruction and repair. Therefore, in addition to increased training of surgeons in surgical techniques, the introduction of implanted mesh with hemostatic ability will have a promising prospect for reducing the bleeding-related concerns.

**Figure 4.**
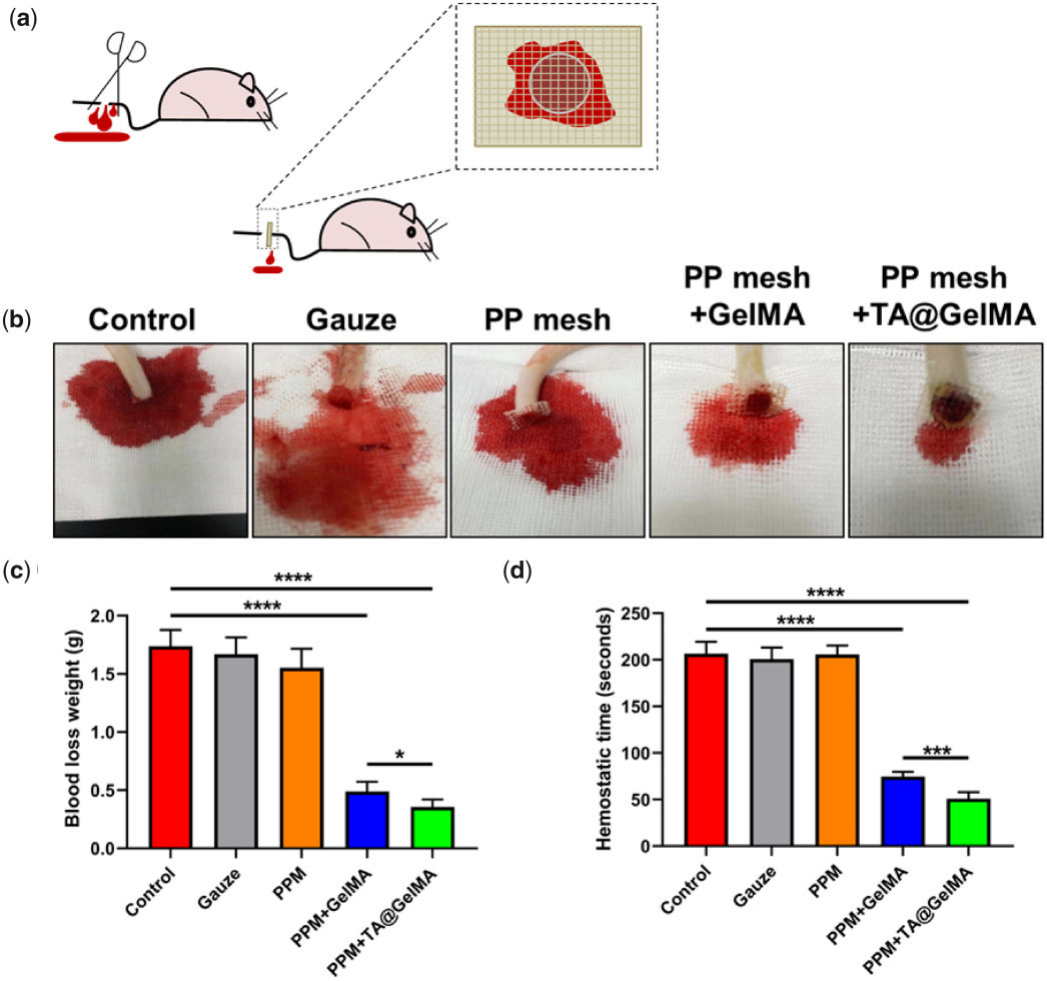
Hemostatic ability assessment. (**a**) Schematic illusion of rat tail amputation model for hemostatic test; (**b**) photographs of bleeding in control, gauze, PP mesh, PP mesh + GelMA and PP mesh + TA@GelMA groups; (**c**) quantitative calculation of blood loss weight of each group; (**d**) quantitative calculation of hemostatic time of each group. *P <* *0.05, **P <* *0.01, ***P <* *0.001 and ****P <* *0.0001.

**Figure 5.**
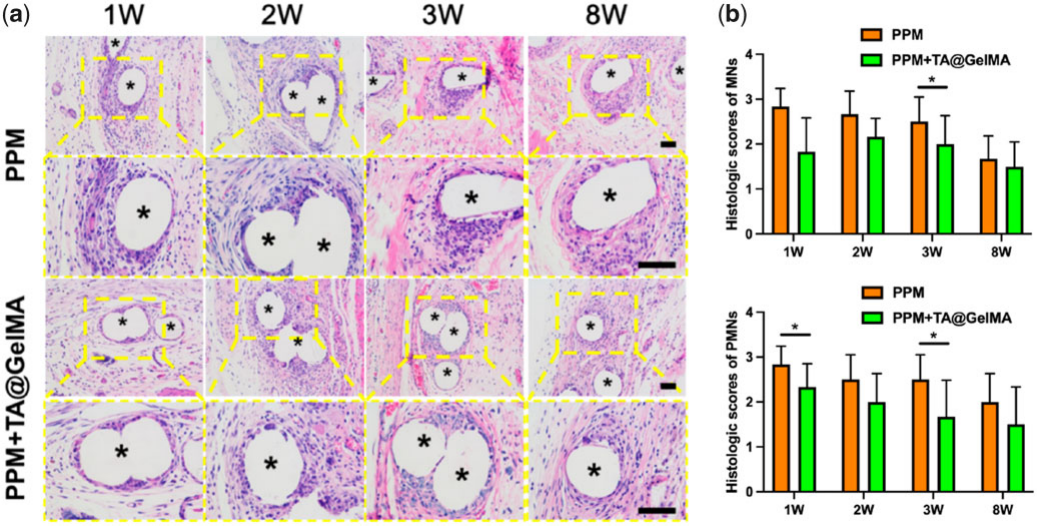
HE staining evaluation of inflammation. (**a**) The HE staining results of defects repaired with PPM and PPM + TA@GelMA at 1, 2, 3 and 8 weeks after implantation (five-pointed asterisks represent monofilaments of PPM); (**b**) histologic scores of MN, PMN. Scale bar, 100 μm. *P <* *0.05.

The hemostatic ability of the hydrogel-mesh complexes was assessed by measuring blood loss weight and hemostatic time in a rat tail amputation model (Fig. 4a). As shown in Fig. 4b, all rats were divided into five groups, and Control group (the group without any treatment was used as a negative control), and gauze, PPM, freeze-dried PPM + GelMA and freeze-dried PPM + TA@GelMA (about 1 cm × 1 cm) were attached to the amputated tail to stop bleeding. Compared with the Control group, the gauze group did not have any significant difference, while the PPM + GelMA and PPM + TA@GelMA groups had significantly less blood loss (Fig. 5c) and significantly faster hemostasis time (Fig. 4d). The blood loss in the PPM + GelMA and PPM + TA@GelMA groups was reduced from 1.738 ± 0.140 g in the control group to 0.489 ± 0.084 and 0.355 ± 0.066 mg, respectively; the hemostatic time was also reduced from more than 3 min in the control group to 74.60 ± 5.18 and 51.00 ± 7.07 s in the PPM + GelMA and PPM + TA@GelMA groups, respectively. The results showed that PPM had no hemostatic effect at all, and the hemostatic mechanism of the gauze group was consistent with that of the Control group; the PPM + GelMA and PPM + TA@GelMA groups had better hemostatic ability than gauze. The freeze-dried hydrogel-mesh complexes with good hydrophilicity and swelling properties could quickly absorb liquid in the blood to form concentrated blood components, which was conducive to coagulation; when the hydrogel-mesh complexes were in contact with blood, the fibrinogen in the blood and other proteins could be stuck to the surface of complexes and stimulated adhesion and aggregation of platelets, activating the coagulation response. Furthermore, the analysis results of Fig. 4c and b showed that compared with the PPM + GelMA group, the PPM + TA@GelMA group had less blood loss (*P *<* *0.01) and faster hemostatic time (*P *<* *0.05). Relevant literature [56, 57] reported that TA could interact with proteins, which was consistent with our result that TA could directly agglutinate GelMA protein in Fig. 1b. Therefore, the improved hemostatic ability of the PPM + TA@GelMA group was the result of the synergistic effect of GelMA hydrogel and TA. TA@GelMA coating may reduce the risk of hematoma after PPM implantation.

### Histological evaluation of inflammation

The optimal POP animal models for POP should be squirrel monkeys and ewes [58–60], which could simulate the pathophysiology of humanity. However, abdominal defect models could be used as a POP model to evaluate the repair effects and host responses of implanted materials [22, 60, 61]. Since most anti-large model animal antibodies are not commercially available, IHC and biochemical assessments involving the use of antibodies cannot be carried out, meaning that parts of repair effects and immune responses cannot be assessed and rodent models such rats can be utilized as surrogate models to avoid these obstacles. Moreover, PPM was initially designed, investigated and used for abdominal hernia repair, and was then gradually applied for the repair of POP, so abdominal repair effects of modified PPM could be used as a reference for POP repair. An expert review [60] has showed that standard laboratory rats subjected to abdominal wall defects have been utilized extensively to compare synthetic implants for POP. In particular, a bilateral abdominal defect model with mesh implantation in a rat had been utilized to investigate the biocompatibility and repair effects of the modified PPMs [22, 62].

In order to directly compare the repair effect of the hydrogel-modified PPM with that of PPM, in our study, a randomized self-paired study was designed to verify the *in vivo* anti-inflammatory and repair effects of TA@GelMA coating on PPM in the same rat with bilateral abdominal defects, where PPM (as the control group) and PPM + TA@GelMA (as the verification group) were randomly allocated to overlay the defect regions. The principle of the randomized self-paired study was also employed in a canine abdominal wall defect repair experiment to directly compare novel biomaterials with PPM [43].

After the POP animal model was established (Supplementary Fig. S1a–d), rats were sacrificed to acquire tissue-mesh complexes for histologic evaluations. It can be concluded from gross histological observation that the PPM + TA@GelMA group showed no serious abnormal tissue encapsulation after 1 week of implantation as free of severe inflammatory response compared to PPM group (Supplementary Fig. S1e). Explanted mesh from women with complications showed poorly regulated inflammation with a fibrous capsule [1]. And there were no exposition, abscess and fold in two groups. Only one case of infection occurred in PPM group. Some literatures reported that TA has a good anti-bacterial effect [35, 39, 63, 64]. However, there were no significant differences between the two groups in terms of anti-infection or anti-bacterial.

HE staining showed that a large number of inflammatory cells were found to wrap the mesh monofilament fibers at the defect areas implanted with materials in each group. At the early stage (1, 2 and 3 week), large amounts of monocytes (MN) and polymorphic megakaryocytes were infiltrated around the monofilament fibers in the PPM group; the monofilament fibers of PPM + TA@GelMA group were also surrounded by polymorphic megakaryocytes merged by multiple macrophages [65], and the number of infiltrated MN was slightly less than that in the PPM group. At the long-term stage (8 weeks), compared with the PPM + TA@GelMA group, there were still numerous mononuclear cells infiltrating around the monofilament fibers in the PPM group, suggesting the persistence of the inflammatory response, which may lead to local chronic inflammation (Fig. 5a). Microscopic evidence of polymorphonuclear cells (PMN), MN was assessed by the scoring system (Supplementary Table S2) described by Badylak [66]. Histological scores in scoring system for MN and polymorphic megakaryocytes were calculated for each group. Overall, all histological scores of MNs and PMNs in the PPM + TA@GelMA group were lower than those in the PPM group at the early and late stages of implantation (Fig. 5b). Compared to PPM group, both scores of MNs at 1 and 3 weeks and scores of PMNs at 3 weeks in PPM + TA@GelMA group were significantly lower (all *P *<* *0.05; Fig. 5b), indicating TA@GelMA coating could significantly mitigate the inflammation response induced by PPM implantation at the early stage. Although PPM could supply strong and stable support for pelvic floor because of its strong mechanical strength and non-degradability, the foreign body reaction (FBR) of PPM, which produced amounts of pro-inflammatory cytokines such as TNF-α at the early stage after implantation [67] and formed a persistent inflammation [5], bothered mesh investigators and clinicians for a long time. Pieces of evidences and reviews stated that predominantly accumulated inflammatory cells were infiltrated around the filaments of the mesh [1, 68]. At the late stage, although no significant differences of PMNs and MNs scores existed between the two groups, PPM + TA@GelMA group scored lower than PPM group in histological evaluation of PMNs and MNs. HE staining results suggested that PPM + TA@GelMA could alleviate the inflammation of PPM at an early stage and maintain a mild chronic inflammatory status.

### Histological evaluation of regenerative capability

**Figure 6.**
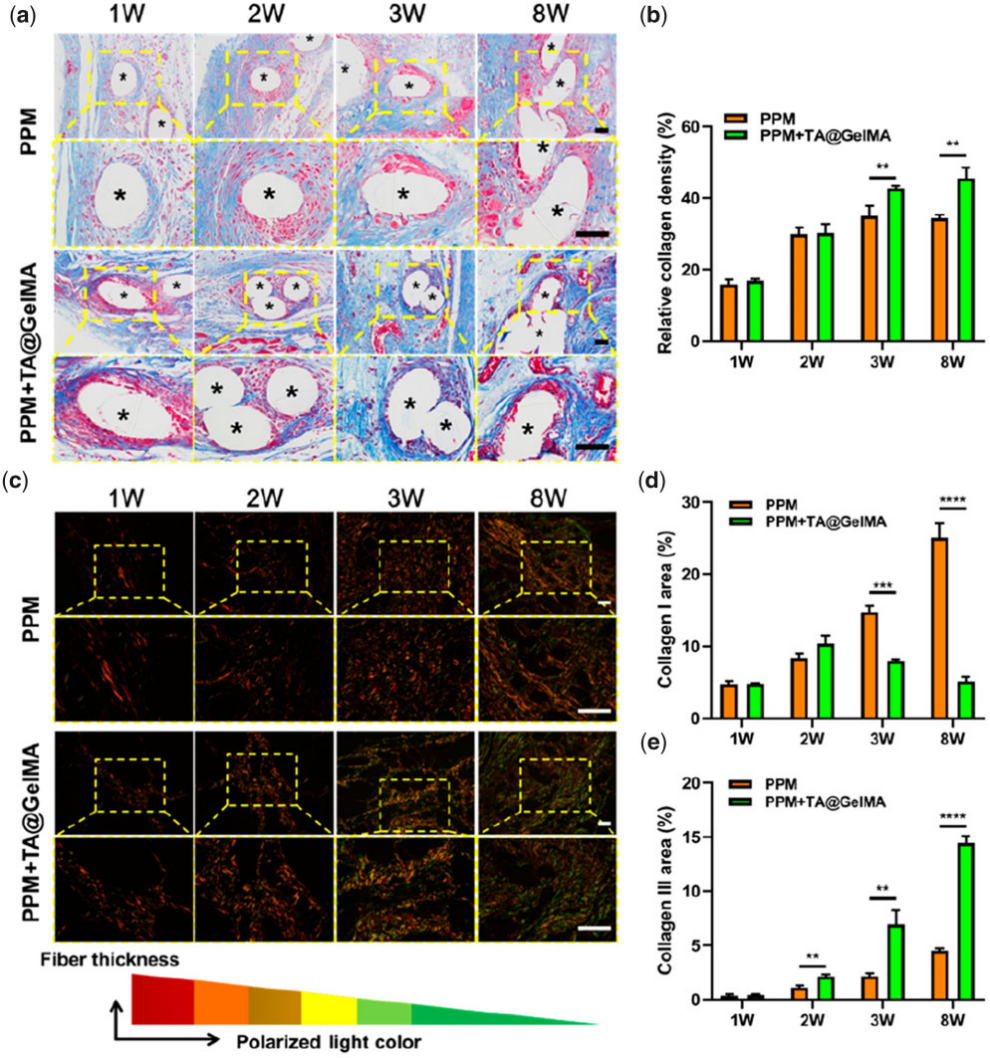
Masson and Sirius staining of collagen regeneration. (**a**) The Masson staining results of collagen regeneration in defects repaired with PPM and PPM + TA@GelMA at 1, 2, 3, 8 weeks after implantation (five-pointed asterisks represent monofilaments of PPM); (**b**) quantitative calculation of relative collagen density of each group; (**c**) the Sirius staining results of collagen subtypes at 1, 2, 3, 8 weeks after implantation. Collagen I was red fibers and Collagen III was green fibers; (**d**) The percentage of Collagen I area; (**e**) the percentage of Collagen III area. Scale bar, 100 μm. **P <* *0.01, ***P <* *0.001 and ****P <* *0.0001.

**Figure 7.**
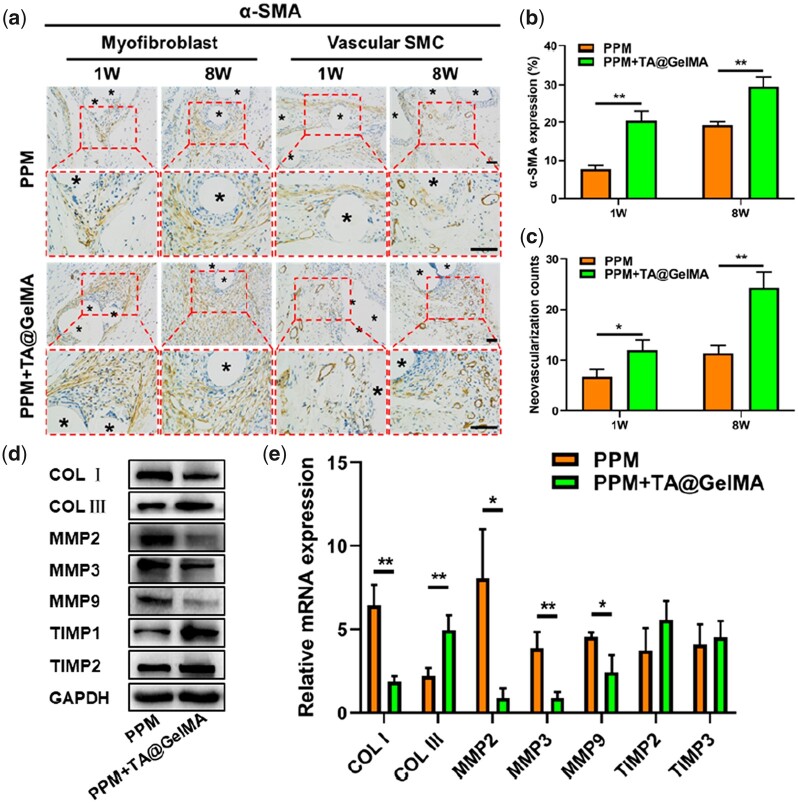
IHC Evaluation of myofibroblast and vascular smooth muscle cell (SMC) evaluation. (**a**) The α-SMA IHC staining results of defects treated with PPM and PPM + TA@GelMA at 1, 8 weeks after implantation (five-pointed asterisks represent monofilaments of PPM); (**b**) the percentage of α-SMA positive expression; (**c**) counts of neovascularization; (**d**) western blotting bands of Collagen I (COL I), Collagen III (COL III), MMPs (MMP2, MMP3, MMP9), tissue inhibitor of metalloproteinase (TIMP2, TIMP2) and glyceraldehyde-3-phosphate dehydrogenase (GAPDH); (**e**) the mRNA expression levels of COL I, COL III, MMP2, MMP3, MMP9, TIMP2, TIMP3. Scale bar, 100 μm. *P <* *0.05, **P <* *0.01.

According to the wound healing process theory, prolongation of the inflammation phase would prolong the process to the proliferative phase and cause delayed wound healing. In this study, the intervention of inflammatory response with anti-inflammation coating at the early stage was more favorable for the integration of mesh and tissue. Compared with the PPM group, PPM + TA@GelMA group better promoted the repair and regeneration of the tissue in the defect area (Figs 6 and 7).

In Fig. 6a, fibrous connective tissues were shown in blue, and the greater the density of collagen fibers, the more intense the blue. Collagen fibers increased further in both groups over time. In the PPM group, collagen fibers did not appear in some defect areas at 1–3 weeks; at 8 weeks, the defect areas were covered, but fibers were low in density and irregular. In the PPM + TA@GelMA group, there were also some defect areas without collagen fibers at 1–2 weeks. At 8 weeks, a large number of fibers occurred in the defect areas and were arranged tightly and regularly, and new blood vessels could even be observed at 3 weeks (Fig. 6b).

Collagen is one of the main components in connective tissues. Type I collagen is a kind of thick fibers and mainly gives tissues tensile properties; the thinner type III collagen has elastic properties. In human prolapsed samples, an increased ratio of type I/III collagen and loss of elastic fibers, and decreased expression of elastin content were found [69]. These changes in the main components of ECM may contribute to the decreased elasticity and impaired mechanical property of pelvic supporting tissues, and the decreased ratio of Collagen III/I may play a key role in the development of POP [69].

To determine the subtype and distribution of collagen fibers, sections were stained with Sirius Red. Under polarized optical microscopy, the thicker type I collagens appeared red or yellow, while the finer type III collagens appeared green (Fig. 6c). As shown in Fig. 6d and e, the density of type I collagen fibers was higher in the PPM group over time, and the type III collagen that improved the elastic strength of the tissue was predominant in the PPM + TA@GelMA group. Western blotting and RT-qPCR experiments were performed on the tissue-mesh complexes of 8 weeks (Fig. 7c and d), and the results also confirmed that the expression of type III collagen was higher in PPM + TA@GelMA than the PPM group. The protein and transcriptome expression levels of MMPs in the PPM group were significantly higher than those in PPM + TA@GelMA group, which were consistent with the collagen density results of Masson staining (Fig. 5a and b). The higher ratio of type III/I collagen in PPM + TA@GelMA group could facilitate the repair of prolapsed tissues.

Previous study demonstrated TGF-β1 had strong correlations of with type III/I collagen ratio [70]. Evidences show TGF-β1 acts as an anti-inflammatory cytokine that mediates the transition from a pro-inflammatory response to a pro-remodeling tissue healing response for ECM regeneration and angiogenesis through TGF-β1/Smad pathways [70–73]. In Supplementary Fig. S3, the TGF-β1 expression level of PPM + TA@GelMA group was significantly higher than that of the PPM group at 2, 3, 8 weeks, indicating TGF-β1 might be involved in the inflammation regulation of TA@GelMA coating for promoting the more increased expression of type III collagen to provide better elasticity and mechanical property for pelvic supporting tissues.

In order to further evaluate the myofibroblast [74] and angiogenesis effect of PPM + TA@GelMA in defect repair, alpha smooth muscle actin (α-SMA) IHC staining was performed on nascent tissue-mesh complexes in the defect area. Interestingly, the α-SMA expression of PPM + TA@GelMA was higher than that of the PPM group (Fig. 7a). The PPM + TA@GelMA group promoted the generation of more myofibroblasts (Fig. 7b), which was beneficial to the repair of the defect tissues [75]. The number of new blood vessels in the PPM + TA@GelMA group was significantly higher than that in the PPM group (Fig. 7c), which indicated that PPM + TA@GelMA had better pro-vascularization properties. CD31 staining was using for detecting neo-angiogenesis [76–78], but there were no vessels around PPM monofilaments at defect area in fact when meshes were implanted and the α-SMA positive vessels could represent the occurrence of neovascularization.

All the above results supported that TA@GelMA coating, which was more cyto-compatible (Fig. 2), could alleviate early stage FBR and promote the tissue repair, thus inducing the ECM and vascular regeneration.

### Regulation of M2 macrophage polarization to promote tissue repair

**Figure 8.**
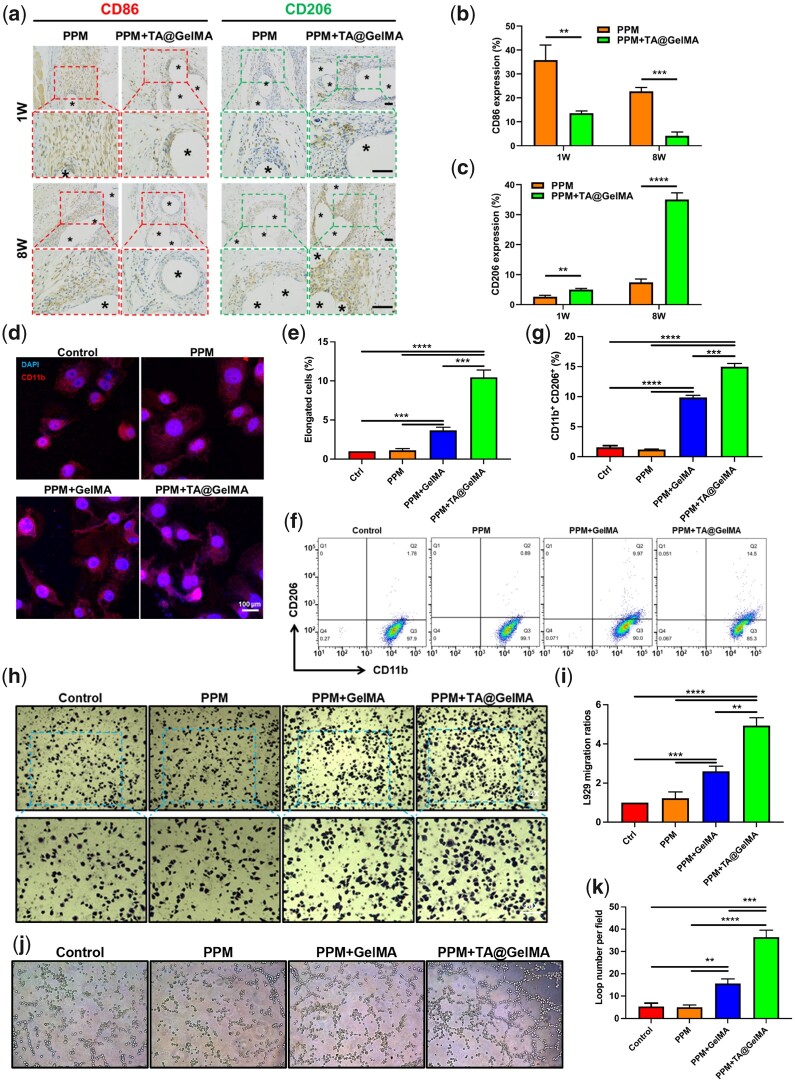
M2 macrophage polarization simulated by PPM + TA@GelMA to promote tissue repair. (**a**) The CD86 and CD206 IHC staining results of defects treated with PPM and PPM + TA@GelMA at 1, 8 weeks after implantation (five-pointed asterisks represent monofilaments of PPM); (**b**) the percentage of CD86 positive expression; (**c**) the percentage of CD206 positive expression; (**d**) the CD11b immunofluorescence staining results of RAW 264.7 macrophages; (**e**) quantitative data of elongated macrophages; (**f**) flow cytometry results of M2-like (CD206 positive) macrophages; (**g**) quantitative data of CD11b^+^ & CD206^+^ macrophages; (**h**) transwell migration results of L929 in M2 macrophage conditioned medium (×40 and ×100 magnification); (**i**) counts of migrated L929 cells; (**j**) tube formation assay of HUVECs on matrigel in treated with M2 macrophage conditioned medium (×100 magnification); (**k**) counts of formed loops. Scale bar, 100 μm. **P <* *0.01, ***P <* *0.001 and ****P <* *0.0001.

Macrophages respond to foreign bodies in the host by phagocytosis [65]. Macrophage phenotype could be a predictor of constructive remodeling following the implantation of meshes [12]. It was observed from H&E staining that TA@GelMA coating reduced the inflammation of PPM and PMNs formed by macrophages responding to foreign meshes (Fig. 5). To further assess the inflammatory status of the defect engraftment areas, macrophages were labeled with IHC staining for CD86 and CD206 at 1 and 8 weeks (Fig. 8a), followed by an analysis of M1 and M2 subtype macrophage expression. As shown in Fig. 8b, CD86-labeled macrophages exhibited higher levels of positive staining in the PPM group, and the percentage of macrophages expressing CD206 in the PPM + TA@GelMA group was significantly higher than that in the PPM group (Fig. 8c), indicating that TA@GelMA coating could effectively alleviate the inflammatory response caused by PPM implantation. At the early stage (1 weeks), TA@GelMA began to regulate the macrophage phenotype with a small population of alternatively activated M2 macrophages infiltrated around the filaments. At the long-term stage (8 weeks), large amounts of anti-inflammatory M2 macrophages were activated and involved in the tissue repair and regeneration.

Overall, PPM with TA@GelMA hydrogel coating could effectively and better benefit collagen regeneration and neo-vascularization by activating M2 macrophages polarization. In our study, all above results showed that the TA@GelMA hydrogel coating could reverse the inflammatory microenvironment (the more infiltrated pro-inflammatory cells and higher ratio of M1/M2) caused by the PPM, increase the number of pro-regenerative M2 macrophages in the defect area, and promote tissue repair and regeneration.

GelMA hydrogel as a kind of degradable material with good bio-compatibility has shown it possesses a bit potential to activate M2 macrophages polarization [79] and inhibit M1 subtypes [80]; however, its limited immunomodulatory capability restricts its further application, and the same for its regenerative capability [15], so it more often works or is designed as a carrier for drug delivery in defects repair [15, 26, 30]. It is worth noting that anti-inflammatory drugs-loaded GelMA hydrogels showed better repair and regeneration effects than GelMA hydrogel [15, 79].

Herein, the regulation of M1/M2 macrophage polarization was spotlighted as a possible mechanism for tissue repair and regeneration as reported in many other studies.

Subsequently, macrophage polarization regulated by PPM + TA@GelMA was investigated by *in vitro* immunofluorescence morphological experiments and flow cytometry of detecting surface markers. Supernatants from meshes or hydrogel-meshes soaking solution were used to incubated with RAW 264.7 macrophages. CD11b (red) and DAPI (blue) was stained for representing cytoplasm/membranes and nuclei respectively. As shown in Fig. 8d, macrophages in Control group (no treatment) appeared round; in contrast, the macrophages in the PPM + GelMA and PPM + TA@GelMA groups had elongated cell shapes, and appeared long pseudopodia from a round shape, showing the typical morphology of M2-subtype macrophages. PPM + TA@GelMA groups induced most M2-like macrophages (Fig. 8e). To further confirm the macrophage polarization status, flow cytometric staining was performed for M2 macrophage surface markers of CD206 and CD11b in different groups (Fig. 8f). The most dual positive staining of CD206 and CD11b could be observed in PPM + TA@GelMA group, followed by PPM + GelMA, PPM and Control groups (Fig. 8). As expected, M2 macrophages polarization was the most significant in PPM + TA@GelMA group, followed by PPM + GelMA group, and the least in PPM group and Control group. Particularly, as shown in Fig. 8e and g, statistically more M2 subtype macrophages were induced in PPM + TA@GelMA group than PPM + GelMA group (*P *<* *0.001). Undoubtedly, TA@GelMA coating has better ability to shift macrophage polarization to M2 phenotype, suggesting the synergic effects of TA with GelMA hydrogel. Furthermore, the results of transwell migration assay and tube formation assay showed the conditioned medium of polarized macrophages in PPM + TA@GelMA group could induce the most L929 fibroblasts to migrate (Fig. 8h and i) and most significantly enhance *in vitro* angiogenesis of HUVECs (Fig. 8j and k). Anti-inflammation M2 phenotype macrophages are known to mediate the tissue regeneration. *In vitro*, the conditioned medium of M2 macrophages in PPM + TA@GelMA group had better chemotactic activity to induce migration of fibroblasts and stimulate the angiogenic capacity of HUVECs. Although RAW 264.7 and HUVECs were of different species origin, macrophages (RAW 264.7) polarized toward M2 subtypes could enhanced the ability of tubule formation of HUVECs as reported [81]. With more migrated fibroblasts and formed blood vascular structures in PPM + TA@GelMA group, it could be speculated that the introduction of TA@GelMA to PPM, which could shift M2 macrophage phenotype, better facilitated the recruitment, migration of cells involved in tissue regeneration. Further studies about the specific cytokines secreted by M2 macrophages in PPM + TA@GelMA group should be performed to identify their important effects on collagen regeneration and neo-angiogenesis. And the molecular mechanism of PPM + TA@GelMA regulating macrophage polarization still needs further studies. Uncovering the mechanisms of materials regulating immune regulation and promoting tissue repair will provide a better basis for the future design and development of regenerative biomaterials.

## Conclusion

In summary, the direct ‘green’ self-crosslinking method (without chemical initiators), which utilized TA and GelMA to form a hydrogel coating on the PPM, completed the dressing modification of the mesh and successfully constructed a new hydrogel-mesh complex (PPM + TA@GelMA) for POP repair. PPM + TA@GelMA exhibited good hydrophilicity, mechanical properties for pelvic pressure and cyto-compatibility. Due to the synergistic effect of TA and GelMA, PPM + TA@GelMA had a hemostatic effect, which was beneficial to intraoperative and postoperative hemostasis or to reduce possible hematoma formation. Further, it was worth noting that the TA@GelMA coating could alleviate the inflammatory response caused by PPM implantation, help the defective tissue repair, promote the collagen fibers regeneration, and trigger neo-angiogenesis. In addition, PPM + TA@GelMA was preliminarily confirmed that could regulate the transformation of macrophages to M2 subtype and participate in the tissue repair process.

Therefore, the authors believe that the method of fabricating this ‘green’ self-crosslinking hydrogel coating and PPM + TA@GelMA will show great potential in the research and treatment of pelvic floor tissue repair.

## Supplementary data

Supplementary data are available at *REGBIO* online.

## Supplementary Material

rbac074_Supplementary_Data

## References

[1] Abhari RE , Izett-Kay ML, Morris HL, Cartwright R, Snelling SJB. Host-biomaterial interactions in mesh complications after pelvic floor reconstructive surgery. Nat Rev Urol 2021;18:725–38. 34545239 10.1038/s41585-021-00511-y

[2] Mangir N , Aldemir Dikici B, Chapple CR, MacNeil S. Landmarks in vaginal mesh development: polypropylene mesh for treatment of SUI and POP. Nat Rev Urol 2019;16:675–89. 31548731 10.1038/s41585-019-0230-2

[3] Alt CD , Benner L, Mokry T, Lenz F, Hallscheidt P, Sohn C, Kauczor HU, Brocker KA. Five-year outcome after pelvic floor reconstructive surgery: evaluation using dynamic magnetic resonance imaging compared to clinical examination and quality-of-life questionnaire. Acta Radiol 2018;59:1264–73. 29409326 10.1177/0284185118756459

[4] Mangir N , Roman S, Chapple CR, MacNeil S. Complications related to use of mesh implants in surgical treatment of stress urinary incontinence and pelvic organ prolapse: infection or inflammation? World J Urol 2020;38:73–80. 30759272 10.1007/s00345-019-02679-w PMC6954150

[5] Elmer C , Blomgren B, Falconer C, Zhang A, Altman D. Histological inflammatory response to transvaginal polypropylene mesh for pelvic reconstructive surgery. J Urol 2009;181:1189–95. 19152931 10.1016/j.juro.2008.11.030

[6] Durst PJ , Heit MH. Polypropylene mesh predicts mesh/suture exposure after sacrocolpopexy independent of known risk factors: a retrospective Case-Control study. Female Pelvic Med Reconstr Surg 2018;24:360–6. 28657987 10.1097/SPV.0000000000000452

[7] Chill HH , Ben Porat L, Winer J, Moss NP, Cohen A, Shveiky D. Infected pelvic hematoma following vaginal hysterectomy with uterosacral ligament suspension for treatment of apical prolapse. Eur J Obstet Gynecol Reprod Biol 2022;271:97–101. 35180514 10.1016/j.ejogrb.2022.02.006

[8] Caveney M , Haddad D, Matthews C, Badlani G, Mirzazadeh M. Short-term complications associated with the use of transvaginal mesh in pelvic floor reconstructive surgery: results from a multi-institutional prospectively maintained dataset. Neurourol Urodyn 2017;36:2044–8. 28407297 10.1002/nau.23231

[9] Wihersaari O , Karjalainen P, Tolppanen AM, Mattsson N, Jalkanen J, Nieminen K. Complications of pelvic organ prolapse surgery in the 2015 finnish pelvic organ prolapse surgery survey study. Obstet Gynecol 2020;136:1135–44. 33156186 10.1097/AOG.0000000000004159

[10] Nolfi AL , Brown BN, Liang R, Palcsey SL, Bonidie MJ, Abramowitch SD, Moalli PA. Host response to synthetic mesh in women with mesh complications. Am J Obstet Gynecol 2016;215:206.e1–206.e8. 27094962 10.1016/j.ajog.2016.04.008 PMC5201165

[11] Darzi S , Urbankova I, Su K, White J, Lo C, Alexander D, Werkmeister JA, Gargett CE, Deprest J. Tissue response to collagen containing polypropylene meshes in an ovine vaginal repair model. Acta Biomater 2016;39:114–23. 27163402 10.1016/j.actbio.2016.05.010

[12] Brown BN , Londono R, Tottey S, Zhang L, Kukla KA, Wolf MT, Daly KA, Reing JE, Badylak SF. Macrophage phenotype as a predictor of constructive remodeling following the implantation of biologically derived surgical mesh materials. Acta Biomater 2012;8:978–87. 22166681 10.1016/j.actbio.2011.11.031 PMC4325370

[13] Ma Y , Zhang Y, Chen J, Li L, Liu X, Zhang L, Ma C, Wang Y, Tian W, Song X, Li Y, Zhu L. Mesenchymal stem cell-based bioengineered constructs enhance vaginal repair in ovariectomized rhesus monkeys. Biomaterials 2021;275:120863. 34139509 10.1016/j.biomaterials.2021.120863

[14] Aghaei-Ghareh-Bolagh B , Mukherjee S, Lockley KM, Mithieux SM, Wang Z, Emmerson S, Darzi S, Gargett CE, Weiss AS. A novel tropoelastin-based resorbable surgical mesh for pelvic organ prolapse repair. Mater Today Bio 2020;8:100081. 10.1016/j.mtbio.2020.100081 PMC7658716 33210083

[15] Qin M , Jin J, Saiding Q, Xiang Y, Wang Y, Sousa F, Sarmento B, Cui W, Chen X. In situ inflammatory-regulated drug-loaded hydrogels for promoting pelvic floor repair. J Control Release 2020;322:375–89. 32243976 10.1016/j.jconrel.2020.03.030

[16] Dahlgren E , Kjølhede P, RPOP-PELVICOL Study Group. Long-term outcome of porcine skin graft in surgical treatment of recurrent pelvic organ prolapse. An open randomized controlled multicenter study. Acta Obstet Gynecol Scand 2011;90:1393–401. 21895613 10.1111/j.1600-0412.2011.01270.x

[17] Robert M , Girard I, Brennand E, Tang S, Birch C, Murphy M, Ross S. Absorbable mesh augmentation compared with no mesh for anterior prolapse: a randomized controlled trial. Obstet Gynecol 2014;123:288–94. 24402595 10.1097/AOG.0000000000000105

[18] Wu C , Zhang Z, He H, Zhou Z, Li H, Tong X. Six-year follow-up outcomes of the P(LLA-CL)/Fg bio-patch for anterior vaginal wall prolapse treatment. Int Urogynecol J 2022. doi: 10.1007/s00192-022-05284-5. 35831453

[19] Hachim D , LoPresti ST, Yates CC, Brown BN. Shifts in macrophage phenotype at the biomaterial interface via IL-4 eluting coatings are associated with improved implant integration. Biomaterials 2017;112:95–107. 27760399 10.1016/j.biomaterials.2016.10.019 PMC5121003

[20] Badiou W , Lavigne J-P, Bousquet P-J, O’Callaghan D, Marès P, de Tayrac R. In vitro and in vivo assessment of silver-coated polypropylene mesh to prevent infection in a rat model. Int Urogynecol J 2011;22:265–72. 21107810 10.1007/s00192-010-1330-y

[21] Parizzi NG , Rubini O, Almeida SHM, Ireno LC, Tashiro RM, Carvalho VHT. Effect of platelet-rich plasma on polypropylene meshes implanted in the rabbit vagina: histological analysis. Int Braz J Urol 2017;43:746–52. 27819759 10.1590/S1677-5538.IBJU.2016.0177 PMC5557452

[22] Faulk DM , Londono R, Wolf MT, Ranallo CA, Carruthers CA, Wildemann JD, Dearth CL, Badylak SF. ECM hydrogel coating mitigates the chronic inflammatory response to polypropylene mesh. Biomaterials 2014;35:8585–95. 25043571 10.1016/j.biomaterials.2014.06.057 PMC5942585

[23] Feola A , Endo M, Urbankova I, Vlacil J, Deprest T, Bettin S, Klosterhalfen B, Deprest J. Host reaction to vaginally inserted collagen containing polypropylene implants in sheep. Am J Obstet Gynecol 2015;212:474.e1–474.e8. 25446700 10.1016/j.ajog.2014.11.008

[24] Hasani-Sadrabadi MM , Sarrion P, Pouraghaei S, Chau Y, Ansari S, Li S, Aghaloo T, Moshaverinia A. An engineered cell-laden adhesive hydrogel promotes craniofacial bone tissue regeneration in rats. Sci Transl Med 2020;12:eaay6853. 10.1126/scitranslmed.aay6853 32161103

[25] Li D , Chen K, Tang H, Hu S, Xin L, Jing X, He Q, Wang S, Song J, Mei L, Cannon RD, Ji P, Wang H, Chen T. A logic-based diagnostic and therapeutic hydrogel with multistimuli responsiveness to orchestrate diabetic bone regeneration. Adv Mater 2022;34:e2108430. 34921569 10.1002/adma.202108430

[26] Wei B , Wang W, Liu X, Xu C, Wang Y, Wang Z, Xu J, Guan J, Zhou P, Mao Y. Gelatin methacrylate hydrogel scaffold carrying resveratrol-loaded solid lipid nanoparticles for enhancement of osteogenic differentiation of BMSCs and effective bone regeneration. Regen Biomater 2021;8:rbab044. 34394955 10.1093/rb/rbab044 PMC8358478

[27] Yang B , Liang C, Chen D, Cheng F, Zhang Y, Wang S, Shu J, Huang X, Wang J, Xia K, Ying L, Shi K, Wang C, Wang X, Li F, Zhao Q, Chen Q. A conductive supramolecular hydrogel creates ideal endogenous niches to promote spinal cord injury repair. Bioact Mater 2022;15:103–19. 35386356 10.1016/j.bioactmat.2021.11.032 PMC8941182

[28] Xu W , Wu Y, Lu H, Zhu Y, Ye J, Yang W. Sustained delivery of vascular endothelial growth factor mediated by bioactive methacrylic anhydride hydrogel accelerates peripheral nerve regeneration after crush injury. Neural Regen Res 2022;17:2064–71. 35142698 10.4103/1673-5374.335166 PMC8848599

[29] Zhang FX , Liu P, Ding W, Meng QB, Su DH, Zhang QC, Lian RX, Yu BQ, Zhao MD, Dong J, Li YL, Jiang LB. Injectable Mussel-Inspired highly adhesive hydrogel with exosomes for endogenous cell recruitment and cartilage defect regeneration. Biomaterials 2021;278:121169. 34626937 10.1016/j.biomaterials.2021.121169

[30] Gu L , Li T, Song X, Yang X, Li S, Chen L, Liu P, Gong X, Chen C, Sun L. Preparation and characterization of methacrylated gelatin/bacterial cellulose composite hydrogels for cartilage tissue engineering. Regen Biomater 2020;7:195–202. 32296538 10.1093/rb/rbz050 PMC7147361

[31] Liu Y , Fan J, Lv M, She K, Sun J, Lu Q, Han C, Ding S, Zhao S, Wang G, Zhang Y, Zang G. Photocrosslinking silver nanoparticles-aloe vera-silk fibroin composite hydrogel for treatment of full-thickness cutaneous wounds. Regen Biomater 2021;8:rbab048. 34513005 10.1093/rb/rbab048 PMC8419525

[32] Yue K , Trujillo-de Santiago G, Alvarez MM, Tamayol A, Annabi N, Khademhosseini A. Synthesis, properties, and biomedical applications of gelatin methacryloyl (GelMA) hydrogels. Biomaterials 2015;73:254–71. 26414409 10.1016/j.biomaterials.2015.08.045 PMC4610009

[33] Abadehie FS , Dehkordi AH, Zafari M, Bagheri M, Yousefiasl S, Pourmotabed S, Mahmoodnia L, Validi M, Ashrafizadeh M, Zare EN, Rabiee N, Makvandi P, Sharifi E. Lawsone-encapsulated chitosan/polyethylene oxide nanofibrous mat as a potential antibacterial biobased wound dressing. Eng Regen 2021;2:219–26.

[34] Zhou X , Saiding Q, Wang X, Wang J, Cui W, Chen X. Regulated exogenous/endogenous inflammation via “Inner-Outer” medicated electrospun fibers for promoting tissue reconstruction. Adv Healthc Mater 2022;11:e2102534. 34989182 10.1002/adhm.202102534

[35] Wu T , Cui C, Fan C, Xu Z, Liu Y, Liu W. Tea eggs-inspired high-strength natural polymer hydrogels. Bioact Mater 2021;6:2820–8. 33718664 10.1016/j.bioactmat.2021.02.009 PMC7903155

[36] Chen YN , Jiao C, Zhao Y, Zhang J, Wang H. Self-assembled polyvinyl alcohol-tannic acid hydrogels with diverse microstructures and good mechanical properties. ACS Omega 2018;3:11788–95. 31459270 10.1021/acsomega.8b02041 PMC6645311

[37] Ninan N , Forget A, Shastri VP, Voelcker NH, Blencowe A. Antibacterial and anti-inflammatory pH-responsive tannic acid-carboxylated agarose composite hydrogels for wound healing. ACS Appl Mater Interfaces 2016;8:28511–21. 27704757 10.1021/acsami.6b10491

[38] Yang Y , Zhao X, Yu J, Chen X, Wang R, Zhang M, Zhang Q, Zhang Y, Wang S, Cheng Y. Bioactive skin-mimicking hydrogel band-aids for diabetic wound healing and infectious skin incision treatment. Bioact Mater 2021;6:3962–75. 33937595 10.1016/j.bioactmat.2021.04.007 PMC8079829

[39] Ahmadian Z , Correia A, Hasany M, Figueiredo P, Dobakhti F, Eskandari MR, Hosseini SH, Abiri R, Khorshid S, Hirvonen J, Santos HA, Shahbazi MA. A hydrogen-bonded extracellular matrix-mimicking bactericidal hydrogel with radical scavenging and hemostatic function for pH-responsive wound healing acceleration. Adv Healthc Mater 2021;10:e2001122. 33103384 10.1002/adhm.202001122

[40] Cui C , Sun S, Wu S, Chen S, Ma J, Zhou F. Electrospun chitosan nanofibers for wound healing application. Eng Regen 2021;2:82–90.

[41] Ouyang Y , Chen R, Chu L, Liang J, Zhang X, Li L, Gao T, Li H, Tong X. Safety and efficacy of a self-developed Chinese pelvic repair system and Avaulta repair system for the treatment of pelvic organ prolapse in women: a multicenter, prospective, randomized, parallel-group study. Medicine (Baltimore) 2020;99:e22332. 32957403 10.1097/MD.0000000000022332 PMC7505298

[42] Yang X , Xiao Y, Zhong C, Shu F, Xiao S, Zheng Y, Xia Z. ABT-263 reduces hypertrophic scars by targeting apoptosis of myofibroblasts. Front Pharmacol 2020;11:615505. 33519480 10.3389/fphar.2020.615505 PMC7840494

[43] Li S , Su L, Li X, Yang L, Yang M, Zong H, Zong Q, Tang J, He H. Reconstruction of abdominal wall with scaffolds of electrospun poly(l-lactide-co caprolactone) and porcine fibrinogen: an experimental study in the canine. Mater Sci Eng C Mater Biol Appl 2020;110:110644. 32204076 10.1016/j.msec.2020.110644

[44] Zhang Q , Qiao Y, Li C, Lin J, Han H, Li X, Mao J, Wang F, Wang L. Chitosan/gelatin-tannic acid decorated porous tape suture with multifunctionality for tendon healing. Carbohydr Polym 2021;268:118246. 34127225 10.1016/j.carbpol.2021.118246

[45] Chen K , Lin Q, Wang L, Zhuang Z, Zhang Y, Huang D, Wang H. An all-in-one tannic acid-containing hydrogel adhesive with high toughness, notch insensitivity, self-healability, tailorable topography, and strong, instant, and on-demand underwater adhesion. ACS Appl Mater Interfaces 2021;13:9748–61. 33591721 10.1021/acsami.1c00637

[46] Zhang D , Lin ZYW, Cheng R, Wu W, Yu J, Zhao X, Chen X, Cui W. Reinforcement of transvaginal repair using polypropylene mesh functionalized with basic fibroblast growth factor. Colloids Surf B Biointerfaces 2016;142:10–9. 26925721 10.1016/j.colsurfb.2016.02.034

[47] Ahmad Z , Salman S, Khan SA, Amin A, Rahman ZU, Al-Ghamdi YO, Akhtar K, Bakhsh EM, Khan SB. Versatility of hydrogels: from synthetic strategies, classification, and properties to biomedical applications. Gels 2022;8:167. 35323280 10.3390/gels8030167 PMC8950628

[48] Yi Y , Xie C, Liu J, Zheng Y, Wang J, Lu X. Self-adhesive hydrogels for tissue engineering. J Mater Chem B 2021;9:8739–67. 34647120 10.1039/d1tb01503f

[49] Zou C-Y , Lei X-X, Hu J-J, Jiang Y-L, Li Q-J, Song Y-T, Zhang Q-Y, Li-Ling J, Xie H-Q. Multi-crosslinking hydrogels with robust bio-adhesion and pro-coagulant activity for first-aid hemostasis and infected wound healing. Bioact Mater 2022;16:388–402. 35415284 10.1016/j.bioactmat.2022.02.034 PMC8965776

[50] Wang M , Hu J, Ou Y, He X, Wang Y, Zou C, Jiang Y, Luo F, Lu D, Li Z, Li J, Tan H. Shape-recoverable hyaluronic acid-waterborne polyurethane hybrid cryogel accelerates hemostasis and wound healing. ACS Appl Mater Interfaces 2022;14:17093–108. 35380771 10.1021/acsami.2c01310

[51] Egorikhina MN , Bronnikova II, Rubtsova YP, Charykova IN, Bugrova ML, Linkova DD, Aleynik DY. Aspects of in vitro biodegradation of hybrid fibrin-collagen scaffolds. Polymers (Basel) 2021;13:3470. 34685229 10.3390/polym13203470 PMC8539699

[52] Birch C , Fynes MM. The role of synthetic and biological prostheses in reconstructive pelvic floor surgery. Curr Opin Obstet Gynecol 2002;14:527–35. 12401983 10.1097/00001703-200210000-00015

[53] Paul K , Darzi S, McPhee G, Del Borgo MP, Werkmeister JA, Gargett CE, Mukherjee S. 3D bioprinted endometrial stem cells on melt electrospun poly ε-caprolactone mesh for pelvic floor application promote anti-inflammatory responses in mice. Acta Biomater 2019;97:162–76. 31386931 10.1016/j.actbio.2019.08.003

[54] Jackson LA , Shi H, Acevedo J, Lee S, Annabi N, Word RA, Florian-Rodriguez M. Effect of gelatin methacryloyl hydrogel on healing of the guinea pig vaginal wall with or without mesh augmentation. Int Urogynecol J 2022;33:2223–32. 34999912 10.1007/s00192-021-05031-2

[55] Shi C , Zhao Y, Hu Q, Gong R, Yin Y, Xia Z. Clinical analysis of pain after transvaginal mesh surgery in patients with pelvic organ prolapse. BMC Womens Health 2021;21:46. 33516228 10.1186/s12905-021-01192-w PMC7847570

[56] Honda Y , Nomoto T, Matsui M, Takemoto H, Kaihara Y, Miura Y, Nishiyama N. Sequential self-assembly using tannic acid and phenylboronic acid-modified copolymers for potential protein delivery. Biomacromolecules 2020;21:3826–35. 32786730 10.1021/acs.biomac.0c00903

[57] Shafreen RMB , Lakshmi SA, Pandian SK, Kim YM, Deutsch J, Katrich E, Gorinstein S. In vitro and in silico interaction studies with red wine polyphenols against different proteins from human serum. Molecules 2021;26:6686. 34771095 10.3390/molecules26216686 PMC8587719

[58] Mori da Cunha M , Mackova K, Hympanova LH, Bortolini MAT, Deprest J. Animal models for pelvic organ prolapse: systematic review. Int Urogynecol J 2021;32:1331–44. 33484287 10.1007/s00192-020-04638-1 PMC8203535

[59] Mansoor A , Curinier S, Campagne-Loiseau S, Platteeuw L, Jacquetin B, Rabischong B. Development of an ovine model for training in vaginal surgery for pelvic organ prolapse. Int Urogynecol J 2017;28:1595–7. 28293789 10.1007/s00192-017-3292-9

[60] Couri BM , Lenis AT, Borazjani A, Paraiso MF, Damaser MS. Animal models of female pelvic organ prolapse: lessons learned. Expert Rev Obstet Gynecol 2012;7:249–60. 22707980 10.1586/eog.12.24 PMC3374602

[61] Jin J , Saiding Q, Wang X, Qin M, Xiang Y, Cheng R, Cui W, Chen X. Rapid extracellular matrix remodeling via gene-electrospun fibers as a “patch” for tissue regeneration. Adv Funct Mater 2021;31:2009879.

[62] Wolf MT , Carruthers CA, Dearth CL, Crapo PM, Huber A, Burnsed OA, Londono R, Johnson SA, Daly KA, Stahl EC, Freund JM, Medberry CJ, Carey LE, Nieponice A, Amoroso NJ, Badylak SF. Polypropylene surgical mesh coated with extracellular matrix mitigates the host foreign body response. J Biomed Mater Res A 2014;102:234–46. 23873846 10.1002/jbm.a.34671 PMC3808505

[63] Shao XH , Yang X, Zhou Y, Xia QC, Lu YP, Yan X, Chen C, Zheng TT, Zhang LL, Ma YN, Ma YX, Gao SZ. Antibacterial, wearable, transparent tannic acid-thioctic acid-phytic acid hydrogel for adhesive bandages. Soft Matter 2022;18:2814–28. 35322837 10.1039/d2sm00058j

[64] Sathishkumar G , Gopinath K, Zhang K, Kang ET, Xu L, Yu Y. Recent progress in tannic acid-driven antibacterial/antifouling surface coating strategies. J Mater Chem B 2022;10:2296–315. 35060581 10.1039/d1tb02073k

[65] Mukherjee S , Darzi S, Paul K, Werkmeister JA, Gargett CE. Mesenchymal stem cell-based bioengineered constructs: foreign body response, cross-talk with macrophages and impact of biomaterial design strategies for pelvic floor disorders. Interface Focus 2019;9:20180089. 31263531 10.1098/rsfs.2018.0089 PMC6597526

[66] Badylak S , Kokini K, Tullius B, Simmons-Byrd A, Morff R. Morphologic study of small intestinal submucosa as a body wall repair device. J Surg Res 2002;103:190–202. 11922734 10.1006/jsre.2001.6349

[67] Prudente A , Favaro WJ, Latuf PF, Riccetto CL. Host inflammatory response to polypropylene implants: insights from a quantitative immunohistochemical and birefringence analysis in a rat subcutaneous model. Int Braz J Urol 2016;42:585–93. 27286125 10.1590/S1677-5538.IBJU.2015.0289 PMC4920579

[68] Kavvadias T , Kaemmer D, Klinge U, Kuschel S, Schuessler B. Foreign body reaction in vaginally eroded and noneroded polypropylene suburethral slings in the female: a case series. Int Urogynecol J Pelvic Floor Dysfunct 2009;20:1473–6. 19727536 10.1007/s00192-009-0974-y

[69] Li Y , Nie N, Gong L, Bao F, An C, Cai H, Yao X, Liu Y, Yang C, Wu B, Zou X. Structural, functional and molecular pathogenesis of pelvic organ prolapse in patient and Loxl1 deficient mice. Aging (Albany NY) 2021;13:25886–902. 34923484 10.18632/aging.203777 PMC8751609

[70] Tennyson L , Rytel M, Palcsey S, Meyn L, Liang R, Moalli P. Characterization of the T-cell response to polypropylene mesh in women with complications. Am J Obstet Gynecol 2019;220:187.e1–187.e8. 30419195 10.1016/j.ajog.2018.11.121 PMC6557122

[71] Kim R , Song BW, Kim M, Kim WJ, Lee HW, Lee MY, Kim J, Chang W. Regulation of alternative macrophage activation by MSCs derived hypoxic conditioned medium, via the TGF-β1/Smad3 pathway. BMB Rep 2020;53:600–4. 33050988 10.5483/BMBRep.2020.53.11.177 PMC7704222

[72] Hou Y , Li J, Guan S, Witte F. The therapeutic potential of MSC-EVs as a bioactive material for wound healing. Eng Regen 2021;2:182–94.

[73] Xin Y , Xu P, Wang X, Chen Y, Zhang Z, Zhang Y. Human foreskin-derived dermal stem/progenitor cell-conditioned medium combined with hyaluronic acid promotes extracellular matrix regeneration in diabetic wounds. Stem Cell Res Ther 2021;12:49. 33422138 10.1186/s13287-020-02116-5 PMC7796620

[74] Koga M , Kuramochi M, Karim MR, Izawa T, Kuwamura M, Yamate J. Immunohistochemical characterization of myofibroblasts appearing in isoproterenol-induced rat myocardial fibrosis. J Vet Med Sci 2019;81:127–33. 30464077 10.1292/jvms.18-0599 PMC6361647

[75] Shinde AV , Humeres C, Frangogiannis NG. The role of α-smooth muscle actin in fibroblast-mediated matrix contraction and remodeling. Biochim Biophys Acta Mol Basis Dis 2017;1863:298–309. 27825850 10.1016/j.bbadis.2016.11.006 PMC5163362

[76] Yin J , Gong G, Sun C, Yin Z, Zhu C, Wang B, Hu Q, Zhu Y, Liu X. Angiopoietin 2 promotes angiogenesis in tissue-engineered bone and improves repair of bone defects by inducing autophagy. Biomed Pharmacother 2018;105:932–9. 30021387 10.1016/j.biopha.2018.06.078

[77] Chen X , He W, Sun M, Yan Y, Pang Y, Chai G. STING inhibition accelerates the bone healing process while enhancing type H vessel formation. FASEB J 2021;35:e21964. 34694030 10.1096/fj.202100069RR

[78] Zhai Y , Schilling K, Wang T, El Khatib M, Vinogradov S, Brown EB, Zhang X. Spatiotemporal blood vessel specification at the osteogenesis and angiogenesis interface of biomimetic nanofiber-enabled bone tissue engineering. Biomaterials 2021;276:121041. 34343857 10.1016/j.biomaterials.2021.121041 PMC8477312

[79] Xu W , Sun Y, Wang J, Wang B, Xu F, Xie Z, Wang Y. Controlled release of silibinin in GelMA hydrogels inhibits inflammation by inducing M2-type macrophage polarization and promotes vascularization in vitro. RSC Adv 2022;12:13192–202. 35520139 10.1039/d2ra00498d PMC9064440

[80] Jiang G , Li S, Yu K, He B, Hong J, Xu T, Meng J, Ye C, Chen Y, Shi Z, Feng G, Chen W, Yan S, He Y, Yan R. A 3D-printed PRP-GelMA hydrogel promotes osteochondral regeneration through M2 macrophage polarization in a rabbit model. Acta Biomater 2021;128:150–62. 33894346 10.1016/j.actbio.2021.04.010

[81] Guo S , Yu D, Xiao X, Liu W, Wu Z, Shi L, Zhao Q, Yang D, Lu Y, Wei X, Tang Z, Wang N, Li X, Han Y, Guo Z. A vessel subtype beneficial for osteogenesis enhanced by strontium-doped sodium titanate nanorods by modulating macrophage polarization. J Mater Chem B 2020;8:6048–58. 32627795 10.1039/d0tb00282h

