# Supplementary material for: Tannic acid-loaded hydrogel coating endues polypropylene mesh with hemostatic and anti-inflammatory capacity for facilitating pelvic floor repair
Source: Regen Biomater. 2022 Sep 26;9:rbac074. doi: 10.1093/rb/rbac074 (PMC9575665; doi:10.1093/rb/rbac074)
Supplement: rbac074_Supplementary_Data [file rbac074_supplementary_data.docx]

| **Supplementary Table 1** The primer sequences for RT-qPCR | |
| --- | --- |
| Gene names | Sequences |
| COL I | F: 5'-AAGGTGACAGAGGCATAAAG-3' |
|  | R: 5'-GGAAGCTGAAGTCATAACCA-3' |
| COL III | F: 5'-CATGATGAGCTTTGTGCAAT-3' |
|  | R: 5'-CTGCTGTGCCAAAATAAGAG-3' |
| MMP2 | F: 5'-TACAGACGGCTACCGCTGGTGT-3' |
|  | R: 5'-AGCGCTGGTGCAGCTCTCAT-3' |
| MMP3 | F: 5'-ACAGACCTGGCCCGTTTCCA-3' |
|  | R: 5'-GCAGGGTGCTGACTGCATCGAA-3' |
| MMP9 | F: 5'-TCACGGACACACAGCTGGCA -3' |
|  | R: 5'-ACCACAGCGCGGTGAACGAA-3' |
| TIMP2 | F: 5'-TATTGTGCCCTGGGACACG-3' |
|  | R: 5'-GTCCATCCAGAGGCACTCATC-3' |
| TIMP3 | F: 5'- ACAGACGCCAGAGTCTCCTA-3' |
|  | R: 5'- ACCTCAAGTCTGTCCGGGTA-3' |
| GAPDH | F: 5'-ATGTGCCGGACCTTGGAAG-3' |
|  | R: 5'-CCTCGGGTTAGCTGAGAGATCA-3' |
| COL: collagen; MMP: matrix metalloenzyme; TIMP: tissue inhibitor of metalloproteinase; GAPDH: glyceraldehyde-3-phosphate dehydrogenase. | |


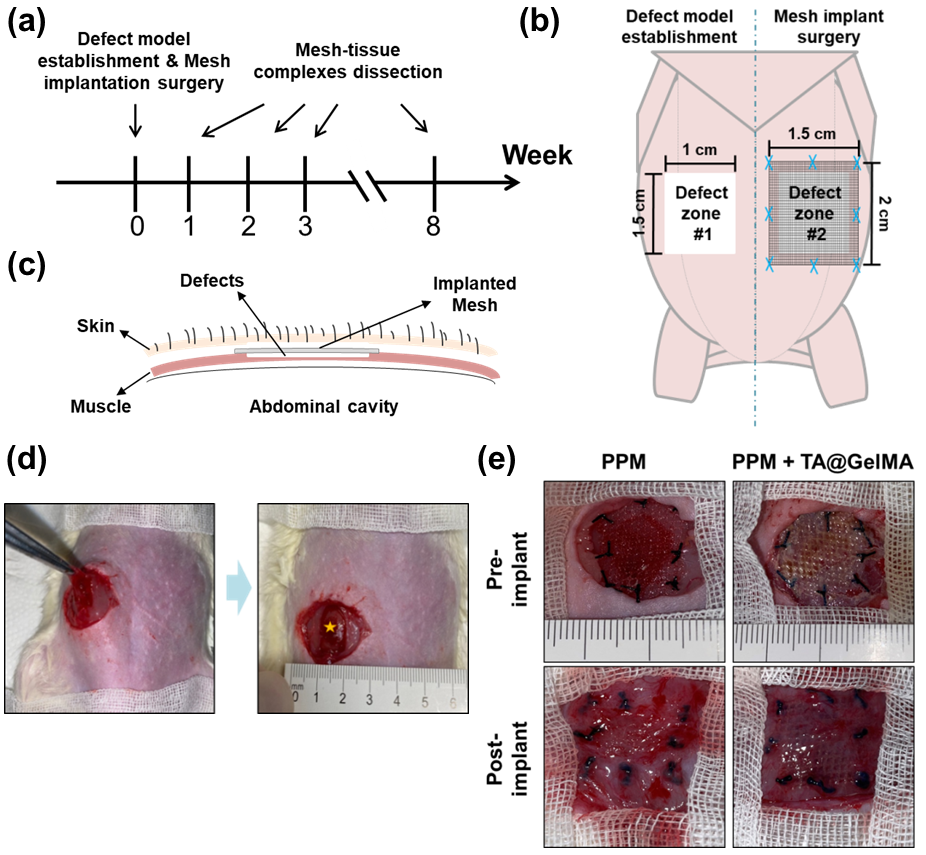


**Supplementary Figure 1** Defect model establishment and gross observation. (a) Timeline of implantation; (b) Schematic illusion of establishing defect model and implanting mesh; (c) Side view of defect implanted with mesh; (d) Images of partial defect model; (e) Gross observation at 2 weeks after implantation surgery.


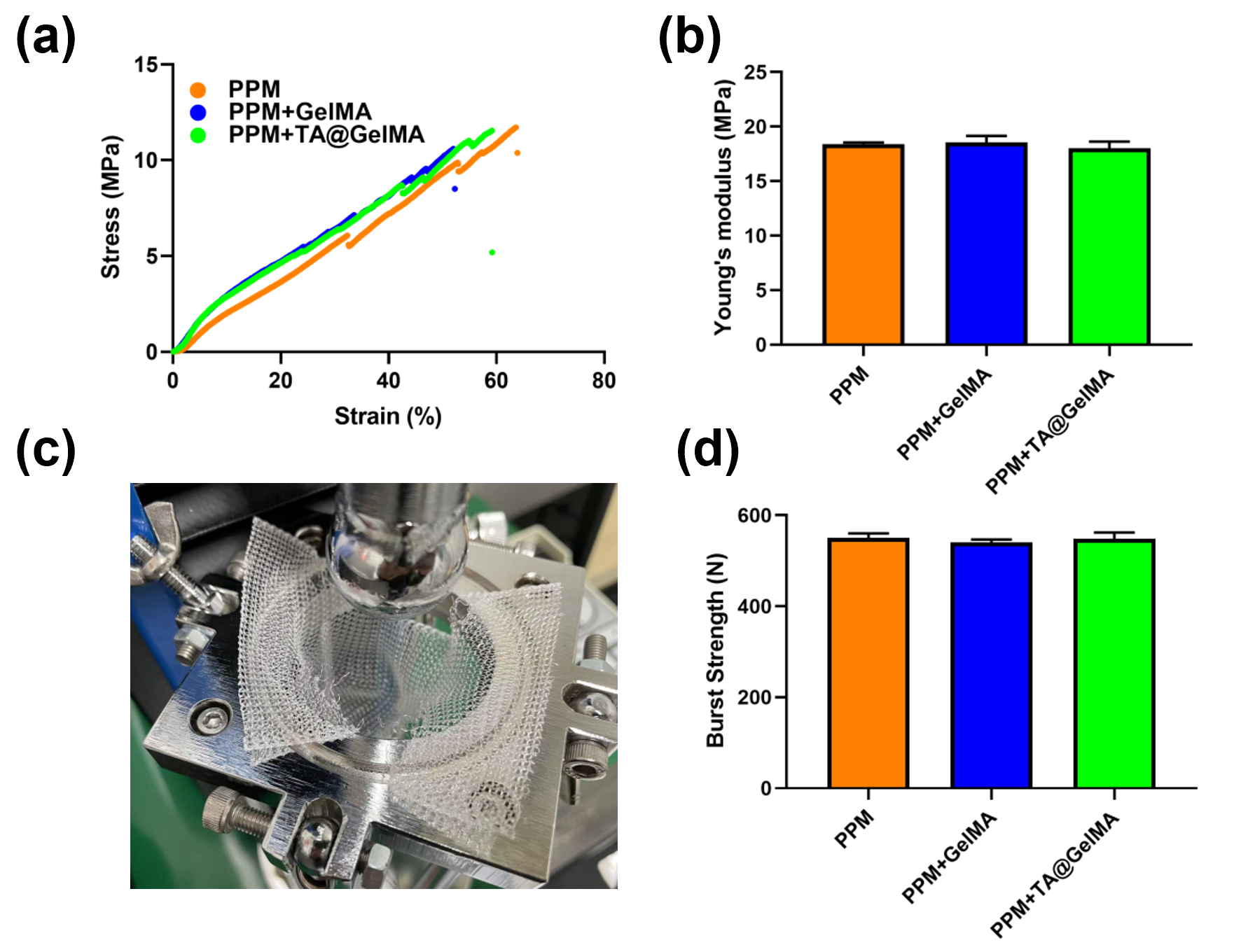


**Supplementary Figure 2:** Mechanical properties of meshes. (a) Stress-strain curve; (b) Calculated result of Young’ s modulus; (c) Ball burst strength test image; (d) Calculated results of burst strength of each group.

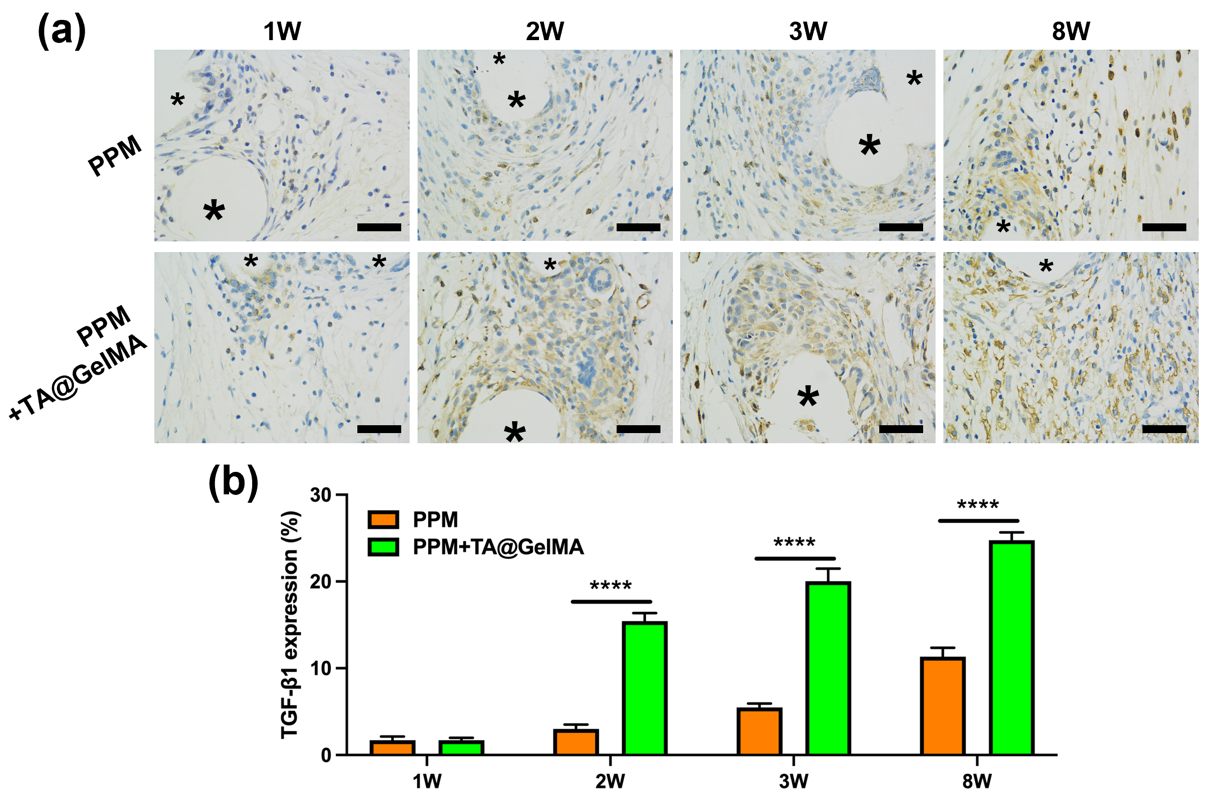


**Supplementary Figure 3:** IHC evaluation of TGF-β1. (a) The TGF-β1 IHC staining results of defects repaired with PPM and PPM+TA@GelMA at 1, 2, 3, 8 weeks (five-pointed asterisks represent monofilaments of PPM); (b) The percentage of TGF-β1 positive expression. Scale bar, 100μm. *****P* < 0.0001.
